# IL-1-driven stromal–neutrophil interactions define a subset of patients with inflammatory bowel disease that does not respond to therapies

**DOI:** 10.1038/s41591-021-01520-5

**Published:** 2021-10-21

**Authors:** Matthias Friedrich, Mathilde Pohin, Matthew A. Jackson, Ilya Korsunsky, Samuel J. Bullers, Kevin Rue-Albrecht, Zoe Christoforidou, Dharshan Sathananthan, Tom Thomas, Rahul Ravindran, Ruchi Tandon, Raphael Sanches Peres, Hannah Sharpe, Kevin Wei, Gerald F. M. Watts, Elizabeth H. Mann, Alessandra Geremia, Moustafa Attar, Oxford IBD Cohort Investigators, Roche Fibroblast Network Consortium, Francesca Barone, Michael Brenner, Christopher D. Buckley, Mark Coles, Andreas P. Frei, Kara G. Lassen, Fiona M. Powrie, Sarah McCuaig, Lloyd Thomas, Elena Collantes, Holm H. Uhlig, Stephen N. Sansom, Alistair Easton, Soumya Raychaudhuri, Simon P. Travis, Fiona M. Powrie

**Affiliations:** 1 Kennedy Institute of Rheumatology, Nuffield Department of Orthopaedics, Rheumatology and Musculoskeletal Sciences, University of Oxford, Oxford, UK; 2 Center for Data Sciences, Brigham and Women’s Hospital, Boston, MA USA; 3 Division of Genetics, Department of Medicine, Brigham and Women’s Hospital, Boston, MA USA; 4 Department of Biomedical Informatics, Harvard Medical School, Boston, MA USA; 5 Program in Medical and Population Genetics, Broad Institute of MIT and Harvard, Cambridge, MA USA; 6 Division of Rheumatology, Inflammation and Immunity, Department of Medicine, Brigham and Women’s Hospital and Harvard Medical School, Boston, MA USA; 7 MRC Weatherall Institute of Molecular Medicine, Radcliffe Department of Medicine, University of Oxford, John Radcliffe Hospital, Oxford, UK; 8 Translational Gastroenterology Unit, NIHR Oxford Biomedical Research Centre, Oxford University Hospitals NHS Foundation Trust, John Radcliffe Hospital, Oxford, UK; 9 Jenner Institute, Nuffield Department of Medicine, University of Oxford, Oxford, UK; 10 The Wellcome Trust Centre for Human Genetics, University of Oxford, Oxford, UK; 11 Department of Paediatrics, John Radcliffe Hospital, Oxford, UK; 12 Old Road Campus Research Building, Department of Oncology, Medical Sciences Division, University of Oxford, Oxford, UK; 13 Centre for Genetics and Genomics Versus Arthritis, Manchester Academic Health Science Centre, University of Manchester, Manchester, UK; 14 Rheumatology Research Group, Institute for Inflammation and Ageing, College of Medical and Dental Sciences, University of Birmingham, Queen Elizabeth Hospital, Birmingham, UK; 15 NIHR Birmingham Biomedical Research Centre, University Hospitals Birmingham NHS Foundation Trust, Birmingham, UK; 16 Roche Pharma Research and Early Development, Immunology, Infectious Diseases and Ophthalmology (I2O) Discovery and Translational Area, Roche Innovation Center Basel, Basel, Switzerland

**Keywords:** Mucosal immunology, Neutrophils, Chemokines, Inflammatory bowel disease, Cytokines

## Abstract

Current inflammatory bowel disease (IBD) therapies are ineffective in a high proportion of patients. Combining bulk and single-cell transcriptomics, quantitative histopathology and in situ localization across three cohorts of patients with IBD (total *n* = 376), we identify coexpressed gene modules within the heterogeneous tissular inflammatory response in IBD that map to distinct histopathological and cellular features (pathotypes). One of these pathotypes is defined by high neutrophil infiltration, activation of fibroblasts and vascular remodeling at sites of deep ulceration. Activated fibroblasts in the ulcer bed display neutrophil-chemoattractant properties that are IL-1R, but not TNF, dependent. Pathotype-associated neutrophil and fibroblast signatures are increased in nonresponders to several therapies across four independent cohorts (total *n* = 343). The identification of distinct, localized, tissular pathotypes will aid precision targeting of current therapeutics and provides a biological rationale for IL-1 signaling blockade in ulcerating disease.

## Abstract (web summary)

Transcriptomic and histological profiling of gut biopsies from multiple independent cohorts of patients with inflammatory bowel disease identifies distinct histopathological, molecular and cellular features associated with treatment response, providing insights for patient stratification and precision therapy.

## Main

Inflammatory bowel diseases are a heterogeneous group of disorders characterized by inflammation throughout the gastrointestinal tract. The etiology involves maladaptation between the host and its intestinal microbiota, a dialog controlled by genetic and environmental factors^1^. The complex nature of IBD is reflected in its clinical phenotypes, encompassing both Crohn’s disease (CD) and ulcerative colitis (UC) and a range of microscopic features such as granulomas, lymphoid aggregates, crypt abscesses and ulcers^2,3^. Treatments for IBD include general immunosuppressants (such as corticosteroids), immunomodulators (such as thiopurines) or biologics (such as antitumor necrosis factor-α, TNF-α) modulating specific inflammatory mediators^4^. However, identification of patients who will respond remains a major challenge.

We previously identified high expression of genes encoding the IL-6 family member oncostatin M (OSM), and its receptor OSMR, in the inflamed intestine of patients with IBD as being associated with nonresponse to anti-TNF therapy^5^. Notably, OSM produced by leukocytes signals primarily into stromal cells such as fibroblasts and endothelial cells. Subsequent transcriptomic studies have associated subsets of fibroblasts, inflammatory mononuclear phagocytes (MNPs), neutrophils and pathogenic T and plasma cells with therapy nonresponse in both UC and CD^6–12^. It remains unknown whether the cellular and molecular hallmarks of treatment response are uniform across patients or whether several different tissular pathologies are linked to therapy failure through distinct mechanisms. Further understanding in those areas is crucial to the design of personalized treatment regimens and new therapeutics for individuals that do not respond to current options.

Here, we explored how individual signatures of tissue inflammation associate with nonresponse to specific medical treatments. Transcriptomic changes linked to therapy nonresponse were found to reflect changes in cellular composition and activation state associated with distinct histological features. These molecular, cellular and histologic definitions of disease provide a basis for rational targeting of existing medications, and an alternative avenue to target inflammation in nonresponsive patients displaying ulceration with fibroblast and neutrophil remodeling.

## Results

### Identification of gene coexpression signatures in inflamed IBD tissue

**Extended Data Fig. 1:**
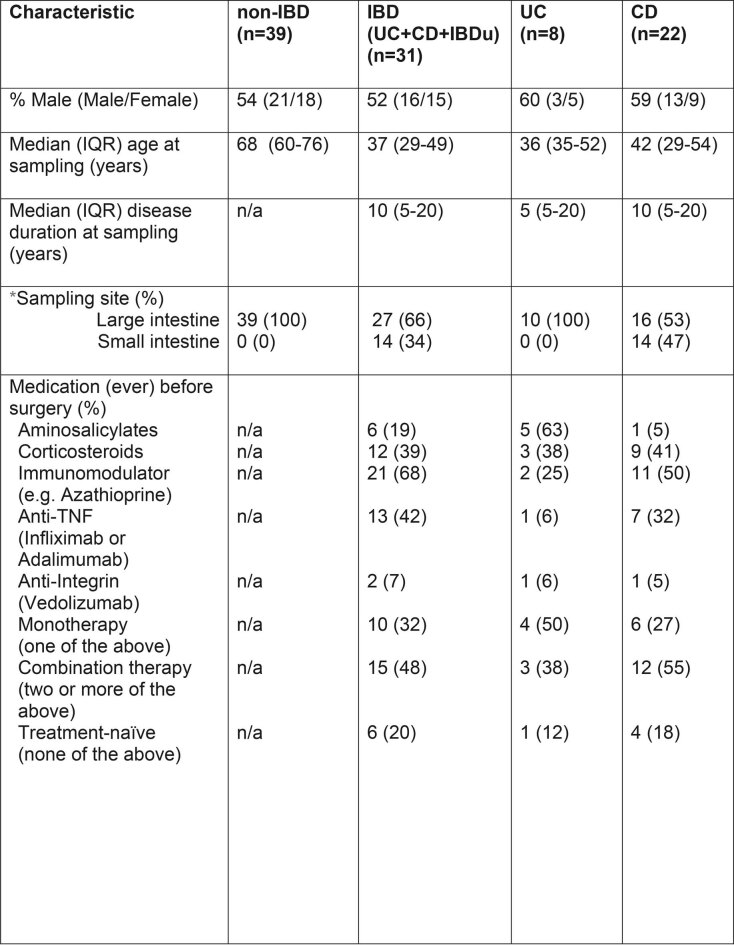
Clinical characteristics of the Oxford IBD patient discovery cohort used in this study. Samples from the discovery cohort consist of surgically removed tissue of UC, CD or IBDu patients (=IBD), as well as surgically removed normal tissue adjacent to colorectal tumors (= non-IBD). IBD, inflammatory bowel disease; CD, Crohn’s Disease; UC, Ulcerative colitis; IQR, interquartile range; n/a, not applicable. * Sampling site percentages are based on the total number of tissues collected (not patient number).

Inflamed tissue from patients with IBD is commonly resected when either medical therapies have failed, when patients opt for elective surgery or when acute complications require emergency surgery. We used such tissues (referred to as difficult-to-treat IBD) as our discovery cohort (Extended Data Fig. 1). Amongst IBD tissue samples (*n* = 41) from the 31 patients in this cohort, 15 were macroscopically uninflamed, including seven samples for which paired uninflamed/inflamed tissue was available. We additionally profiled unaffected, nontumour tissue (*n* = 39) collected from 39 patients with colorectal cancer (CRC) and undergoing surgery as non-IBD controls. Bulk RNA-sequencing (RNA-seq) was used to generate whole-tissue gene expression profiles across all samples (*n* = 41 IBD and *n* = 39 non-IBD; the‘discovery cohort’).

**Fig. 1:**
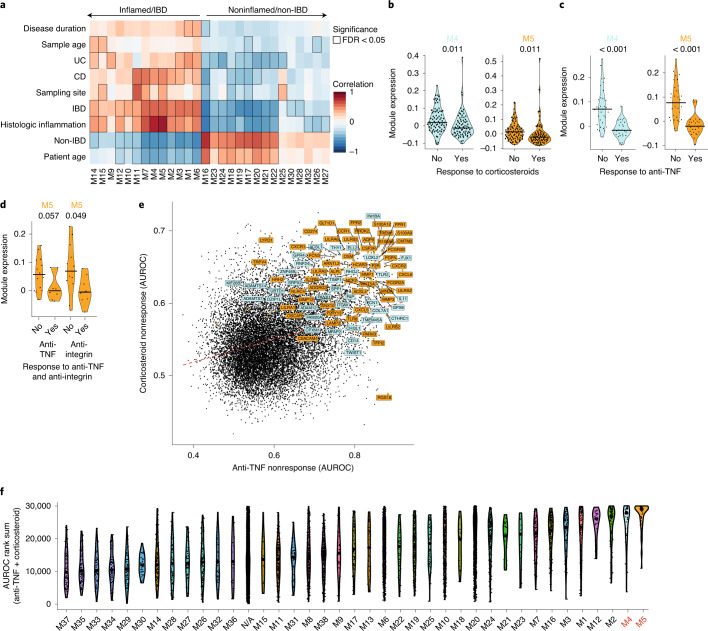
Identification of gene coexpression signatures of inflammation associated with patient nonresponse to multiple different IBD therapies. **a**, Pearson correlation between module eigengenes and clinical and histologic metadata in IBD (*n* = 41) and non-IBD (*n* = 39, normal adjacent to CRC) tissues within the discovery cohort; all modules/features with at least one significant association are shown; bordered squares indicate significant correlations (false-discovery rate (FDR) *P* < 0.05, asymptotic two-tailed *P* values estimated from Pearson coefficients). Exact *P* values are given in Supplementary Table 1. **b**–**d**, Expression of modules M4 and M5 (eigengene value) in nonresponders and responders before the start of administration of either corticosteroid (**b**^10^, *n* = 206 patients), anti-TNF (**c**,**d**^15,16^, *n* = 61 patients) or monthly anti-integrin therapy (**d**^16^, *n* = 20 patients) (two-tailed Mann–Whitney *U*-test, FDR adjusted *P* values; these were post hoc to ANOVA comparisons across the various treatment regimens in ref. ^16^ (**d**). Exact FDR adjusted *P* values (**c**): M4, 2.7 × 10^−6^; M5, 4.7 × 10^−7^. **e**, Performance (area under the receiver operator curve (AUROC)) of individual genes for prediction of nonresponse to corticosteroid (*y* axis) and anti-TNF (*x* axis) therapy; genes in M4 and M5 are labeled and highlighted turquoise and orange, respectively. **f**, Violin plots showing gene rank based on their predictive power (AUROC) for response to both anti-TNF and corticosteroid therapy, comparing all modules as detected in the WGCNA analysis. Combined ranks represent the sum of each gene’s ranks in the separate corticosteroid and anti-TNF analyses (their ranks on the *x* and *y* axes in **e**).

**Extended Data Fig. 2:**
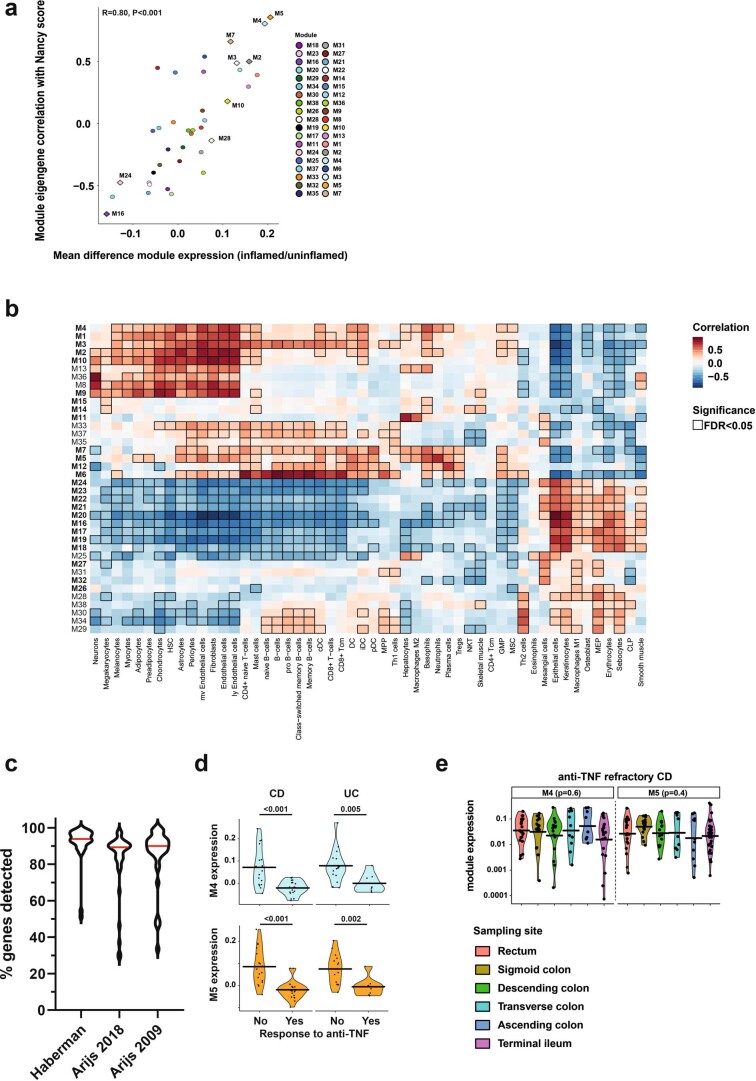
Module associations with inflammation and response to therapy. a) Scatterplot of the module expression difference between inflamed and uninflamed tissues paired from the same patients versus the correlation of the module with the Nancy score across all IBD and non-IBD tissues. Points highlighted with a diamond indicate a significant difference in two-tailed paired t-tests between inflamed/uninflamed tissue (FDR p < 0.1). b) Heatmap of module eigengene – cell type correlations; cell types were deconvoluted from whole tissue expression data using *xCell*. Modules highlighted in bold were fund to be associated with histologic inflammation. c) Percentage of genes within each of the n = 38 modules that were detectable in the publicly available datasets^10,15,16^. Red horizontals in violins indicate the median. d) M4/M5 module expression (eigengene) in patients with CD (n = 37) and UC (n = 24) before anti-TNF therapy start (GSE16879, horizontal bars indicate geometric mean, two-tailed Mann Whitney U test P values are given; exact P-values are 0.0026 and 7.8×10^−6^ for M4 and M5 CD comparisons, respectively). e) M4/M5 module expression (eigengene) in n = 87 patients with CD after anti-TNF therapy failure (GSE100833, horizontal bars indicate geometric mean, P values of comparisons across all sites are given, Kruskall-Wallis test).

To identify sets of genes reflective of distinct biological processes, we applied weighted gene correlation network analysis (WGCNA) to cluster coexpressed genes across all tissue samples. This identified 38 modules of highly coexpressed genes (M1–M38) (Supplementary Table 1). We correlated the expression (module eigengene) of these modules with sample characteristics, clinical phenotypes and histologic (microscopic) inflammation (Nancy index^13^); 28 modules were significantly associated with at least one of these measures (Fig. 1a). Modules were found to have dichotomous associations with traits: about half had significant positive correlations with histologic inflammation while the remainder had significant negative associations (Fig. 1a). Age appeared to have associations similar to inflammation, but this was an artifact of the older nature of the non-IBD samples used as controls. In a paired analysis of only inflamed and uninflamed IBD tissue samples from the same patients (*n* = 7), the difference in expression of a module between tissue pairs remained highly correlated with the module’s association with histologic inflammation (Nancy index) (*R* = 0.8, *P* < 0.001; Extended Data Fig. 2a), confirming that these modules reflected inflammatory processes.

To determine whether the gene coexpression patterns detected reflect changes in the cell type composition of patient tissues, we applied in silico cell type deconvolution analysis to the RNA-seq data of our discovery cohort (*xCell*^14^). Correlating predicted cell type scores with module expression (eigengenes) (Extended Data Fig. 2b), modules positively correlated with histologic inflammation (Fig. 1a) were associated with signatures of stromal cells (for example, fibroblasts), B lymphocytes and plasma cells, T lymphocytes (for example, CD8^+^ T cells) and granulocytes (for example, neutrophils). Modules negatively correlated with histologic inflammation were predicted to reflect epithelial and smooth muscle cells. These results suggest that the coexpression patterns associated with inflammation were, at least in part, driven by differences in cellular composition.

### Coexpression signatures are associated with patient therapy response

To determine whether inflammation-associated modules are relevant to treatment outcomes, we projected the modules onto whole-tissue gene expression data derived from prospective studies of response to anti-TNF, corticosteroid or anti-integrin therapy^10,15,16^. At least 79% of the genes within each module could be identified in the three datasets, enabling accurate quantification within them (Extended Data Fig. 2c and Supplementary Table 2). The expression of 15 modules was significantly (adjusted *P* < 0.05) higher or lower in nonresponders to anti-TNF before treatment (Supplementary Table 3). Seven and two modules, respectively, were significantly higher or lower in nonresponders to corticosteroid and anti-integrin therapy (Supplementary Table 3).

Strikingly, across all three therapy response datasets, modules M4 and M5 were consistently amongst the strongest associations with nonresponse in pretreatment samples (Supplementary Table 3 and Fig. 1b–d). This overall trend of increased expression in nonresponders was significant in meta-analyses of both M4 (*P* = 0.0025, standardized mean difference (SMD) = 0.87, 95% confidence interval (CI) = 0.31–1.44) and M5 (*P* = 0.0123, SMD = 0.88, 95% CI = 0.19–1.58) across the different treatments. Importantly, this association remained when clinical subtypes of IBD (Extended Data Fig. 2d) and intestinal location (Extended Data Fig. 2e) were analyzed separately.

To determine whether the associations with nonresponse are uniform across the genes in modules M4 and M5 or are driven by a small number of them, we compared the contribution of genes by ranking their individual power to predict nonresponse to anti-TNF and corticosteroid therapies. Again, the majority of genes from modules M4 and M5 were among the top predictors of nonresponse to both anti-TNF and corticosteroid therapy relative to genes in other modules (Fig. 1e,f and Supplementary Table 4). Thus, M4 and M5 reflect a coordinated shift in the expression of all their constituent genes in relation to therapy nonresponse across multiple IBD medications.

### Coexpression signatures are represented by histopathologic features

**Fig. 2:**
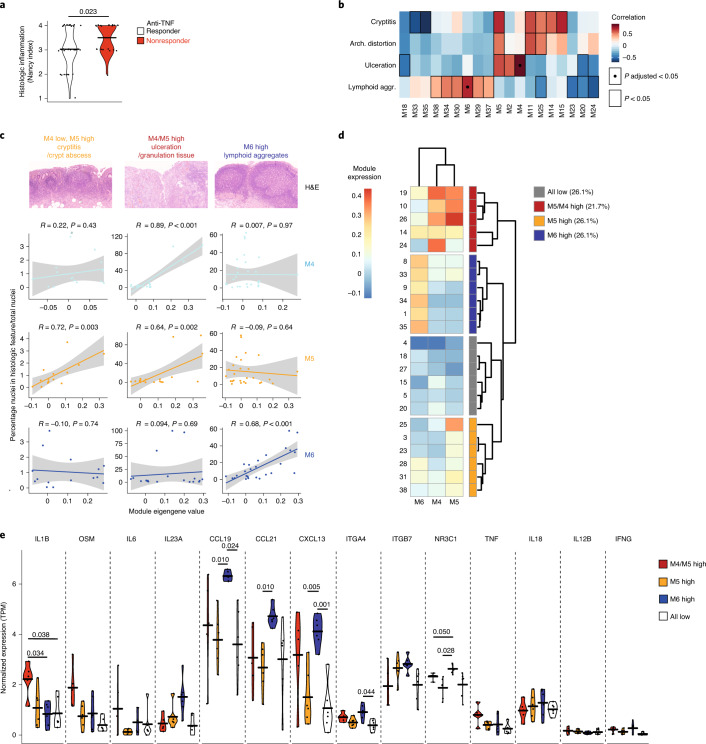
Coexpression modules linked with therapy nonresponse represent distinct histopathologic features. **a**, Nancy histologic scores in responders (*n* = 35 UC) and nonresponders (*n* = 21 UC) to anti-TNF therapy within 3 months before the start of treatment (horizontal bars indicate geometric mean, two-tailed Mann–Whitney *U*-test *P* value is given). **b**, Heatmap of correlations between module eigengene expression and histological features quantified across tissues from IBD patients in the discovery cohort (where histological features could be quantified). Nominally significant associations (*P* < 0.05) are indicated by borders, and FDR significant (FDR *P* < 0.05) associations are indicated by dots; *P* values represent two-tailed probabilities of Pearson correlation coefficients. Exact *P* values can be obtained when rerunning the analysis on https://github.com/microbialman/IBDTherapyResponsePaper. **c**, Scatter plots showing eigengene expression of M4, M5 and M6 versus selected quantified histologic features in tissue samples from patients in the discovery cohort with IBD (linear model fit, error bands show 95% CI; *P* values represent two-tailed probabilities of Pearson correlation coefficients). **d**, Classification of M4/M5-high-tissue (*n* = 5), M5 only-high-tissue (*n* = 6), M6-high-tissue (*n* = 6) and M4/M5/M6 (*n* = 6)-low-tissue patients in the discovery cohort, based on hierarchical clustering of module eigengene values from inflamed tissue samples. **e**, Normalized expression (TPM) of cytokine and therapeutic target genes that were reliably (in >50% of samples) detected in the discovery cohort. The expression of these genes is compared in the M4/M5-high tissue (red), M5 only-high tissue (orange), M6-high tissue (blue) and M4/M5/M6-low tissue (gray). Horizontal lines indicate the median, and *P* values (two-tailed Wilcoxon rank-sum test, adjusted for multiple testing) for each comparison are given if significant (*P* < 0.05). Arch., architectural; aggr., aggregates.

**Extended Data Fig. 3:**
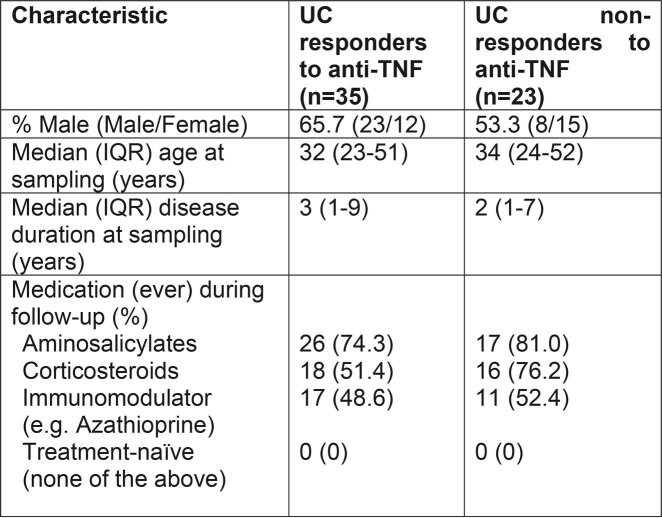
Clinical characteristics of the Oxford UC patient cohort of response to anti-TNF therapy. Response to therapy in this UC patient cohort was defined as stopping anti-TNF therapy (Infliximab or Adalimumab) within 12 months of start, for reason of non response (patients that stopped therapy for convenience, switch to biosimilar, intolerance, or anti-drug antibodies were not considered). Nancy histologic scores and UCEIS endoscopic scores, as well as the other characteristics, within 3 months before the start of anti-TNF therapy are shown. UC, Ulcerative colitis; IQR, interquartile range; UCEIS, Ulcerative Colitis Endoscopic Index of Severity.

**Extended Data Fig. 4:**
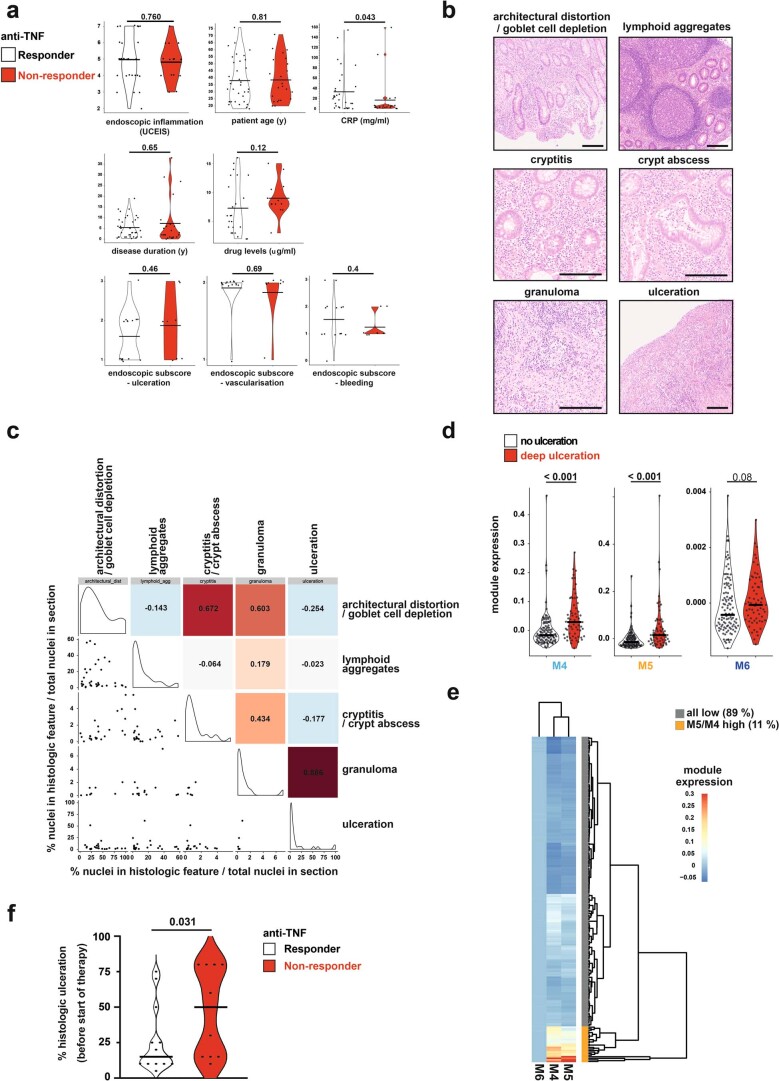
Correlations of histopathologic measures and patient response to therapy. a) Clinical and endoscopic measures in responders (n = 35) and non-responders (n = 21) to anti-TNF therapy before the start of treatment (see Extended Data Fig. 3 for cohort details; horizontal bars indicate geometric mean, two-tailed Mann Whitney U test P values are given; some measures were not reported for all patients, resulting in less datapoints). b) Exemplary images of the various pathological features quantified on H&E histology in resected tissue from IBD patients. Scale bar 200 µm. c) Correlation plot of histological features, quantified as the % of nuclei within the feature area relative to the nuclei with the total section area. Numbers and colours in upper right corner indicate the Pearson correlation coefficient; histograms on diagonal show the value distribution of the features within IBD patient tissues; scatter plots in the lower left corner show the individual datapoints. d) Violin plots of eigengene expression of M4, M5 and M6 in inflamed tissues of IBD patients with (n = 61) or without (n = 111) deep ulceration observed in a replication cohort of paediatric CD and UC; horizontal lines indicate median and adjusted two-tailed Wilcoxon signed rank test P-values are given; exact P-values are 1.4×10^−4^ and 1.1×10^−6^ for M4 and M5 comparisons, respectively. e) Classification of M4/M5 high and M4/M5 low patients in the paediatric replication cohort (n = 172), based on hierarchical clustering on module eigengene values. f) Percent ulceration on biopsies collected within 3 months before anti-TNF therapy start (subcohort of Extended Data Fig. 3), as semi-quantitatively (0-25-50-75-100) scored by a blinded consultant histopathologist (n = 12 responders, n = 10 non responders). Two-tailed Mann-Whitney U test P-values are given. Therapy response was defined as described in Extended Data Fig. 3.

In addition to being highly expressed in tissues from therapy nonresponders, modules M4 and M5 also demonstrated the strongest correlation with histologic inflammation (Nancy index) in the discovery cohort^13^ (Fig. 1a). Using an additional clinical cohort of Oxford UC patients (Extended Data Fig. 3), we confirmed that the Nancy index (Fig. 2a), but not other clinical or endoscopic measures (Extended Data Fig. 4a), is higher in nonresponders to anti-TNF therapy before the start of treatment.

Given the foregoing, we postulated that the observed gene coexpression patterns might also reflect histopathologic features associated with IBD (Extended Data Fig. 4b). First, we examined the correlation of quantified histopathologic features with each other (Extended Data Fig. 4c). The only strong positive correlations observed were between cryptitis/crypt abscess and architectural distortion/goblet cell depletion, as well as several associations with granulomas, although there were few cases where granulomas were detected. We then looked for correlations between module expression and histologic feature scores from tissue where both were available (*n* = 36). Although several nominally significant associations were observed between modules and various histopathological features (Fig. 2b), only positive correlations between M4/ulceration and M6/lymphoid aggregates remained significant after adjusting for multiple testing (*P* adjusted < 0.05; Fig. 2b). Notably, the relation of these two inflammation-associated modules was almost orthogonal, each correlating with only one of the features (Fig. 2c). Despite not reaching significance after correction, M5 also correlated strongly with both ulceration and cryptitis/crypt abscesses (Fig. 2c). We confirmed the associations of M4 and M5 with ulceration in an independent pediatric cohort (*n* = 172) containing inflamed tissues from patients with UC or CD^17,18^ (Extended Data Fig. 4d). In that dataset, 11% of all patients with IBD showed high M4/M5 tissue expression (Extended Data Fig. 4e). Similar to our dataset, M6 expression was not significantly different by ulceration status in the pediatric cohort, although we noted that overall M6 expression was also much lower in those biopsy samples (Discussion). In line with the association of M4/M5 with therapy nonresponse, we were able to demonstrate that the extent of histologic ulceration is significantly higher in nonresponders to anti-TNF therapy before the start of treatment (Extended Data Fig. 4f; subcohort of the UC patient cohort used in Extended Data Fig. 3).

The almost orthogonal relation of M4/M5/ulceration with M6/lymphoid aggregates suggested that these may represent distinct underlying inflammatory processes dominant in a given patient’s tissue. To investigate this, we grouped patients by unsupervised clustering on module M4/M5/M6 expression to determine the relative proportion of patients belonging to these groups. This yielded four groups: M4/M5 high expression (21.7% of patients), M6 high expression, M5-only high expression and M4/M5/M6 low expression (each 26.1% of patients) (Fig. 2d). The M4/M5-high group displayed significantly increased expression of *IL1B* compared to the remainder of the patients (Fig. 2e, red). However, neither *ITGA4*/*ITGB7* (encoding integrin subunits targeted by anti-integrin therapy), *N3RC1* (targeted by corticosteroids) nor *TNF* (targeted by anti-TNF) expression was increased in the tissue of these patients (Fig. 2e). By contrast, high expression of module M6 was linked to increased expression of *ITGA4* and *N3RC1*, as well as *CCL19, CCL21* and *CXCL13* but not *TNF* (Fig. 2e, blue). Patients high in M5 expression only did not demonstrate significant changes in cytokine/therapeutic target signatures (Fig. 2e, orange). Therefore patient responses to specific treatments might be determined by the nature of the inflammatory pathology in the tissue.

### High M4/M5 expression reflects neutrophil infiltrates, fibroblast activation and epithelial cell loss

**Fig. 3:**
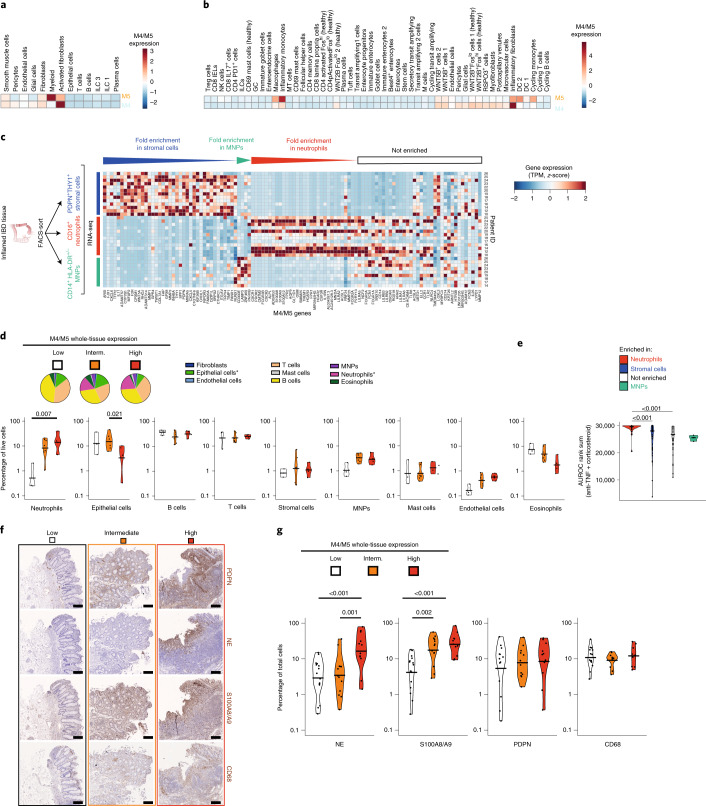
High expression of modules M4 and M5 reflects neutrophil infiltrates, activated fibroblasts and epithelial cell loss. **a**,**b**, Expression of modules M4 and M5 in cell clusters detected by scRNA-seq in tissues of patients with CD^6^ (**a**) and UC^7^ (**b**). **c**, Heatmap of the levels of expression (TPM values, *z*-score, Manhattan distance clustering) of all genes contained within M4 and M5 in THY1^+^PDPN^+^ stromal cells, CD16^hi^ neutrophils and CD14^+^HLA-DR^±^ MNPs, FACS-sorted from *n* = 13, *n* = 12 and *n* = 9 inflamed IBD patient tissues, respectively. Genes are ordered by log fold change of significant enrichment (*P* adjusted < 0.05, two-tailed Wald test; Supplementary Table 5) in a cell type. **d**, FACS cell type percentages in tissue isolates from patients with IBD, classified into low (white, *n* = 3), intermediate (interm.) (orange, *n* = 7) and high (red, *n* = 4) expression of M4/M5 (Extended Data Fig. 3e). Pie-charts (* denotes significantly different between groups, post hoc two-way ANOVA adjusted *P* values) show medians across samples, and violin plots individual samples. **e**, Violin plots showing the combined rank of genes based on their predictive power (AUROC) for response to both anti-TNF and corticosteroid therapy, comparing genes significantly enriched in neutrophils, stromal cells, MNPs or neither (Supplementary Table 5). Horizontal lines indicate the median; adjusted *P* values are given (two-tailed Wilcoxon test; exact *P* values are 2.7 × 10^−5^ and 2.1 × 10^−4^ for neutrophil versus nonspecific and neutrophil versus stroma, respectively). **f**, Illustrative (of samples quantified in **g**) IHC staining (DAB, counterstain hematoxylin) of PDPN, NE, S100A8/A9 and CD68 in serial sections of tissues from patients with IBD classified as low, intermediate or high for M4/M5 whole-tissue gene expression (Extended Data Fig. 3f). Scale bars, 200 µm. **g**, Automated quantification (percentage of positively stained cells of total cells detected in inflamed areas) of IHC stainings shown in **f**. Each staining was quantified on inflamed tissue sections with low (*n* = 15), intermediate (*n* = 13) and high (*n* = 12) M4/M5 whole-tissue gene expression (Extended Data Fig. 3f). Post hoc two-way, two-tailed ANOVA adjusted *P* values are given, where significant.

In silico cell type deconvolution showed that M4 and M5 were predominantly associated with stromal cells including fibroblasts and granulocytes, such as neutrophils (Extended Data Fig. 2b). Projection of modules M4 and M5 onto single-cell transcriptomic datasets derived from inflamed and noninflamed CD^6^ and UC patient tissue^7^ suggested that module M4 reflects the presence of ‘activated/inflammatory fibroblasts’ whereas module M5 reflects ‘myeloid cells/inflammatory monocytes’ (Fig. 3a,b).

**Extended Data Fig. 5:**
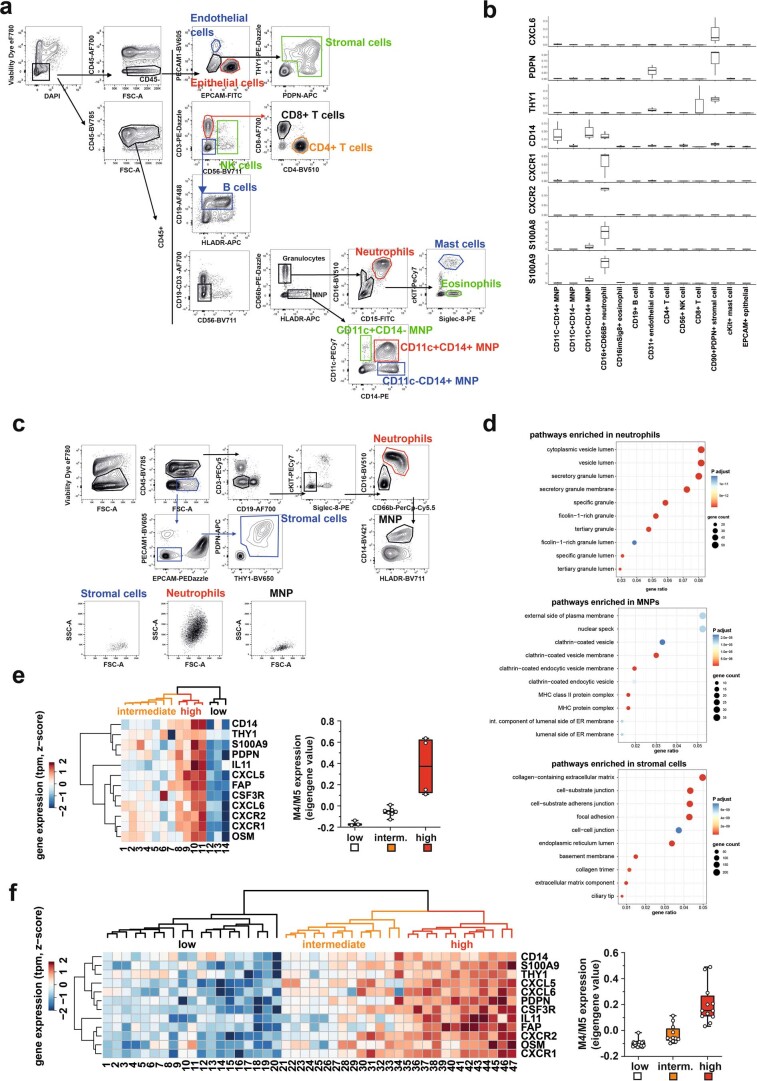
FACS sorting and transcriptomic analysis of cellular subsets. a) Representative gating strategy of FACS sorting hematopoietic and non-hematopoietic cell populations from non-IBD and IBD patient tissue. b) Normalised gene expression (qPCR, relative to *RPLP0* expression) of selected genes from M4 and M5 in cell populations sorted as in a) from n = 3 intestinal tissues of n = 3 patient with IBD; box and whisker plots display median, upper and lower quartiles, and range. c) FACS-gating strategy for sorting of neutrophils, stromal cells and MNPs from tissue samples of IBD patients. d) Gene set enrichment analysis using Gene Ontology (GO) Cellular Components pathway terms, based on all genes significantly enriched (p adjusted <0.05, |log2 fold change | > 2; two-tailed randomisation test) in either neutrophils MNPs or stromal cells (Supplementary Table 5); Exact P-values can be obtained when re-running the analysis on https://github.com/microbialman/IBDTherapyResponsePaper.e + f) Heatmaps of whole tissue gene expression of selected genes that are representative (=highly correlative) of M4 and M5 expression (qPCR, z-score transformed gene expression values); unsupervised clustering (Manhattan) distinguishes subgrouping into M4/M5 low, intermediate and high samples; the box-plots on the right show the eigenvalues of all detected genes on a per patient basis; box and whisker plots display median, upper and lower quartiles, and range. The respective heatmaps refer to tissue samples used for FACS analysis (n = 14, e) and IHC analysis (n = 47, f) as shown in Fig. 3d, f and g.

**Extended Data Fig. 6:**
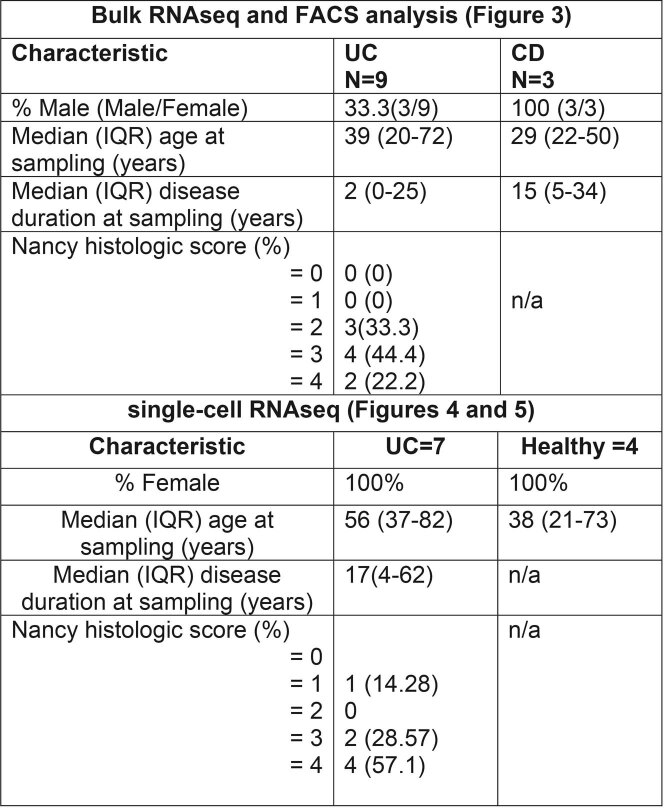
Clinical characteristics of the IBD patients used for RNAseq and FACS analysis. Clinical characteristics of the IBD patient cohorts used for the transcriptomic and FACS analysis. UC, Ulcerative colitis; IQR, interquartile range; UCEIS, Ulcerative Colitis Endoscopic Index of Severity.

Gene expression analysis of FACS-sorted cell populations from inflamed tissue from patients with IBD confirmed that several M4/M5 genes were highly expressed in CD16^hi^ neutrophils and podoplanin (PDPN)^+^THY1^+^ stromal cells (Extended Data Figs. 5a,b and 3c; see Extended Data Fig. 6 for patient cohort details). Pathway analysis on genes upregulated in either cell type (see Supplementary Table 5 for differential gene expression analysis) demonstrated that neutrophils were enriched in antimicrobial and tissue-toxic granule biology when compared to MNPs that were mostly defined by genes belonging to the antigen presentation pathway (Extended Data Fig. 5d). Stromal cells were enriched in many genes assigned to extracellular matrix pathways (Extended Data Fig. 5d). Of all differentially expressed genes between cell types, 35, 28 and 4% of all genes contained in M4/M5 were significantly enriched in stromal cells, neutrophils and MNPs, respectively (Supplementary Table 5 and Fig. 3c). The enrichment of many M4 and M5 genes in sorted neutrophils explained the high correlation of modules M4 and M5 with the Nancy index (Fig. 1a), which is weighted by the abundance of neutrophils^13^.

To assess whether changes in whole-tissue gene expression reflect changes in cellular numbers, we used flow cytometry to test whether neutrophil and fibroblast counts correlated with M4 and M5 tissue expression (see Extended Data Fig. 5c,e for gating strategy and classification of tissues by M4/M5 expression). The percentage of neutrophils was significantly increased (up to tenfold) in M4/M5-high tissues while the percentage of stromal cells remained unchanged (Fig. 3d). Additionally, epithelial cells were significantly decreased in M4/M5-high tissues (Fig. 3d). Neutrophils accounted for up to 38% of total live cells in the M4/M5-intermediate and -high groups, whilst the percentage of MNPs was much lower (<5%) (Fig. 3d). Furthermore, M4/M5 genes significantly enriched in neutrophils and stromal cells, but not in MNPs, demonstrated the highest predictive power for nonresponse to anti-TNF and corticosteroids (Fig. 3e).

We next quantified the presence of neutrophils, stromal cells and MNPs in situ in resected inflamed tissue from patients with IBD (Fig. 3f). Again, inflamed IBD tissues with high expression of M4 and M5 (see Extended Data Fig. 5f for classification) demonstrated a higher percentage of neutrophil elastase (NE)- and calprotectin (S100A8/A9)-positive cells, but not PDPN-positive stromal cells or CD68^+^ MNPs (Fig. 3g).

High M4/M5 expression in whole tissue thus reflects ulceration characterized by a predominance of neutrophil infiltration, expression of genes characteristic of activated fibroblasts and loss of epithelial cells.

### High M4/M5 expression represents neutrophil-attracting fibroblasts and endothelial/perivascular cell expansion

**Fig. 4:**
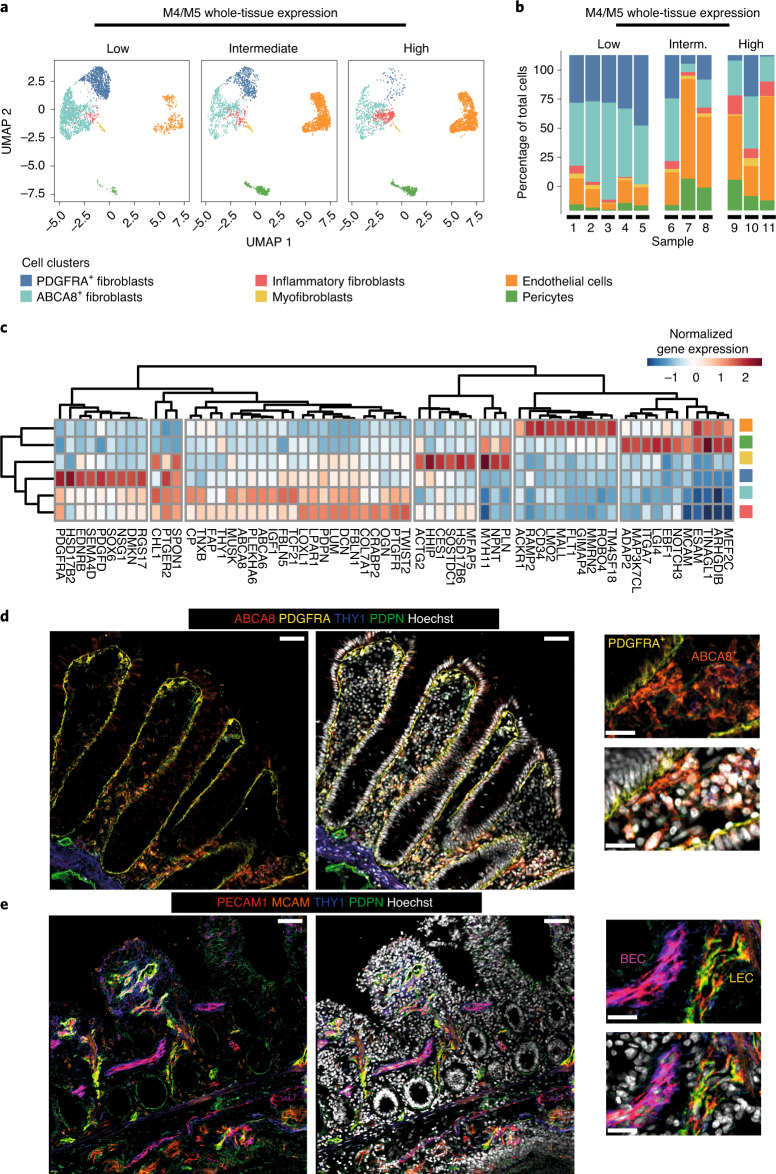
Stromal architecture of the large and small intestine in health and disease. **a**, UMAP of stromal clusters identified by Harmony in stromal compartments, FACS-sorted from healthy donors and tissue from patients with IBD and low (*n* = 5), intermediate (*n* = 3) and high (*n* = 3) M4/M5 whole-tissue gene expression (Extended Data Fig. 4a). **b**, Percentage of total stromal cells among cell type clusters in M4/M5-low, -intermediate and -high tissue. **c**, Heatmap of selected markers of each cellular cluster shown in **a**, as identified by Harmony. Expression values are normalized log_2_ fold changes (Wald statistic $\frac{{\beta _g}}{{\sigma _g}}$) from DESeq2 analyses (where *ß*_*g*_ is log_2_ fold change for gene *g*, and *σ*_*g*_ is estimated standard error of log_2_ fold change for gene *g*). **d**, Immunofluorescent staining of THY1 (blue), PDPN (green), ABCA8 (red) and PDGFRA (yellow) to visualize the localization of fibroblast subsets in resected tissue from patients with IBD (noninflamed areas). Images are representative of stainings in *n* = 8 tissues from *n* = 4 patients. Scale bars, 50 µm (20 µm in zoomed images). **e**, Immunofluorescent staining of THY1 (blue), PDPN (green), PECAM1 (red) and MCAM (orange) to visualize the localization of vascular (endothelial) and perivascular cells (noninflamed areas). Images are representative of staining in *n* = 8 tissues from *n* = 4 patients. Scale bars, 50 µm (20 µm in zoomed images). BEC, blood endothelial cells; LEC, lymphatic endothelial cells.

**Extended Data Fig. 7:**
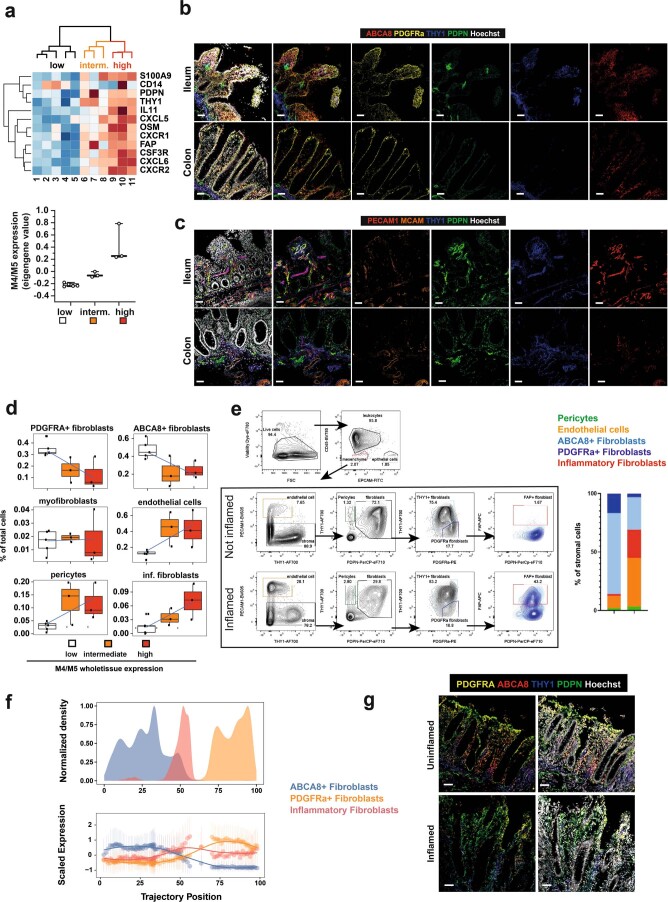
Single-cell transcriptomic and immunofluorescent profiling of patient samples. a) Heatmap of whole tissue gene expression of selected genes that are representative of (highly correlated with) M4 and M5 expression (qPCR, z-score transformed gene expression values); unsupervised clustering (Manhattan) groups samples into M4/M5 low (n = 5 patients), intermediate (n = 3 patients) and high (n = 3 patients) from the set of IBD patients whose samples were profiled by single cell RNA sequencing; the box-plots (lower panel) show the eigenvalues of all detected genes on a per patient basis; data are presented as median values + /- range. b) Immunofluorescent staining of ABCA8 (red), PDGFRA (yellow), THY1 (blue), Podoplanin (PDPN, green) and nuclei (Hoechst, grey) in ileum and colon of resected tissue from IBD patients (not inflamed) (Images representative of stainings in n = 8 tissues of n = 4 patients). c) Immunostaining of PECAM1 (red), MCAM (orange) THY1 (blue), Podoplanin (PDPN, green) and nuclei (Hoechst, grey) in ileum and colonic resected tissue from IBD patients (not inflamed) (Images representative of stainings in n = 8 tissues of n = 4 patients). d) Box plot showing the proportion of the cell types in M4/M5 low (n = 5 patients), intermediate (n = 3 patients) and high groups (n = 3 patients), as detected by scRNAseq; box and whisker plots display median, upper and lower quartiles, and range. e) FACS analysis of live stromal cells (CD45-, EPCAM-) in resected tissue from an IBD patient (adjacent not inflamed and inflamed tissue). Gates for endothelial cells (PECAM1*+*), pericytes (THY + , PDPN-), ABCA8 + fibroblasts (THY1 high, PDGFRa low), PDGFRA + fibroblasts (PDGFRA high, THY1 low) and inflammatory fibroblasts (FAP*+*) are shown. f) Pseudotime analysis of ABCA8 + , PDGFRA + and inflammatory fibroblasts in the single-cell dataset. Cell densities (top row) or canonical markers (bottom) are shown along the trajectory, binned to 100 uniform-density windows (each window has the same number of cells). g) Representative immunofluorescent stainings of PDGFRA (yellow) and ABCA8 (red) staining on fibroblasts in paired inflamed and uninflamed samples of the same IBD patient (Images representative of stainings in n = 8 tissues of n = 4 patients).

Since we did not observe a change in stromal cell numbers in M4/M5-high tissues (Fig. 3d,g), we hypothesized that the detected stromal signatures could have arisen from altered activation states (including the upregulation of PDPN). We applied scRNA-seq to EPCAM^–^CD45^–^ intestinal stromal cells from endoscopic biopsies of inflamed tissue from patients with UC (*n* = 7) and tissue from healthy donors (*n* = 4) (see Extended Data Fig. 6 for patient cohort details), and compared tissues with low, intermediate and high M4/M5 expression (Fig. 4a,b and Extended Data Fig. 7a). As expected, tissues from all healthy donors were M4/M5 low, as well as the tissue from one patient with IBD who had a low histologic inflammation score (Nancy score = 1). We integrated all single-cell datasets, accounting for interpatient and technical batch effects^19^, and identified six stromal clusters. These were assigned to endothelial cells (*ACKR1*^*+*^*CD34*^*+*^), pericytes (*NOTCH3*^+^*MCAM*^*+*^), myofibroblasts (*MYH11*^hi^*ACTG2*^*+*^) and three clusters of fibroblasts: *PDGFRA*^hi^*PDPN*^lo^*SOX6*^+^ (PDGFRA^+^) fibroblasts, *PDGFRA*^lo^*PDPN*^lo^*ABCA8*^hi^ (ABCA8^+^) fibroblasts and *CD90*^hi^*PDPN*^hi^*PDGFRA*^lo^*ABCA8*^lo^*FAP*^+^ ‘inflammatory’ fibroblasts, based on the top differentially expressed markers and previously described annotations^6,7,20^ (Fig. 4c and Supplementary Table 6). *PDPN* was expressed by myofibroblasts and all three fibroblast clusters, with the highest expression found in inflammatory fibroblasts. *THY1* (CD90) was highly expressed in pericytes and inflammatory fibroblasts, but at lower levels in ABCA8^+^ fibroblasts (Fig. 4c).

In situ localization of stromal cells in noninflamed large (colon) and small (ileum) intestinal tissue confirmed that PDGFRA^+^ and ABCA8^+^ fibroblasts represent two spatially distinct fibroblast subsets (Fig. 4d and Extended Data Fig. 7b). Costaining of PDPN and THY1 with markers of fibroblasts (PDGFRA, ABCA8), pericytes (MCAM) and endothelial cells (PECAM1) confirmed that multiple stromal subsets express these markers to varying extents (Fig. 4d,e and Extended Data Fig. 7c,d). Notably, THY1 formed a gradient of staining intensity from the perivascular niche toward the lamina propria (as recently described in ref. ^21^), being expressed by both ABCA8^+^ fibroblasts and MCAM^+^ pericytes, as well as by cells in the muscular layer of the submucosa (Fig. 4d,e and Extended Data Fig. 7b,c).

In the single-cell dataset, the percentage of inflammatory fibroblasts, pericytes and endothelial cells was increased in the M4/M5-intermediate and -high patient groups at the expense of ABCA8^+^ and PDGFRA^+^ fibroblasts (Fig. 4a,b and Extended Data Fig. 7d). FACS analysis verified that PECAM1^+^ endothelial cell and PDPN^+^FAP^+^ inflammatory fibroblast frequencies were increased within the stromal compartment in inflamed compared to noninflamed adjacent tissue (Extended Data Fig. 7e).

**Fig. 5:**
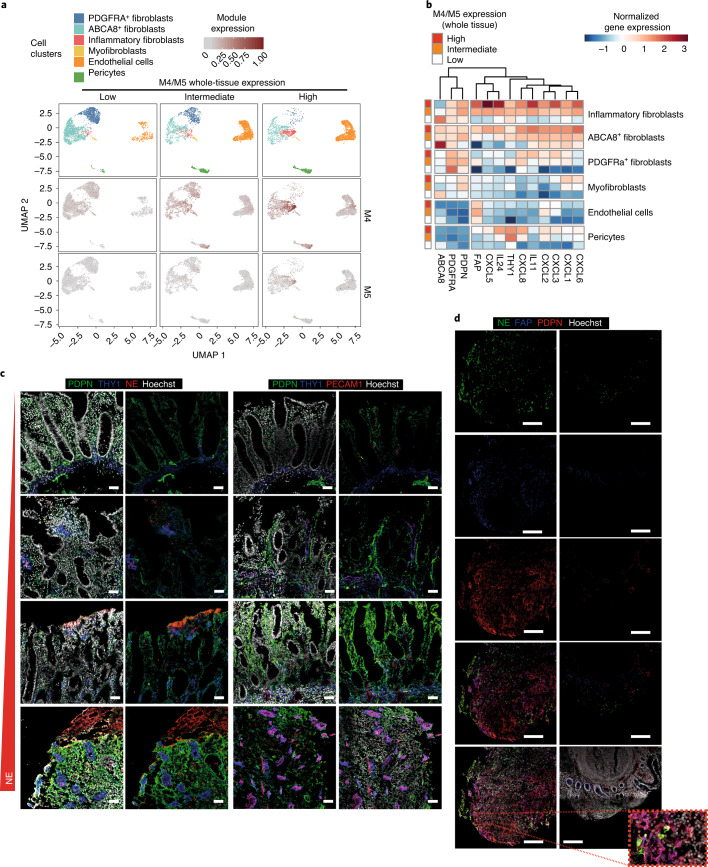
M4/M5 gene expression is associated with neutrophil-attracting fibroblasts and endothelial and perivascular cell expansion. **a**, UMAP of stromal single-cell profiles showing the different stromal clusters as in Fig. 4a for comparison (top), and the expression level of M4 (middle) and M5 (bottom) genes in these clusters. **b**, Heatmap showing normalized gene expression of the top differentially expressed genes between M4/M5 expression levels within each cell cluster. Expression values are normalized log_2_ fold changes (Wald statistic $\frac{{\beta _g}}{{\sigma _g}}$) from DESeq2 analyses. **c**, Staining of NE or PECAM1 (red), THY1 (blue) and PDPN (green) in *n* = 4 tissues from patients with IBD and with varying grades of neutrophil infiltration. Images are representative of stainings in *n* = 8 tissues from *n* = 4 patients. Scale bars, 50 µm. **d**, Staining of NE (green), FAP (blue) and PDPN (red) in paired inflamed (deep ulcer) and noninflamed tissues. Images are representative of staining in *n* = 8 tissues from *n* = 4 patients. Scale bars, 200 µm (20 µm in zoomed image insert).

To determine which of those clusters contributed most to M4/M5 expression, we projected the modules onto our scRNA-seq data. Notably, the highest expression of M4 was detected in the inflammatory fibroblast cluster, suggesting the emergence of this cell cluster as an underlying process in M4/M5-high IBD patient tissue (Fig. 5a). Within M4/M5-high tissues, genes encoding neutrophil-targeting CXCR1/CXCR2 ligands *CXCL1*, *CXCL2*, *CXCL3*, *CXCL5*, *CXCL6* and *CXCL8* were significantly more highly expressed in inflammatory fibroblasts in comparison to other clusters (Fig. 5b and Supplementary Table 7). Within the cluster of inflammatory fibroblasts, *PDPN*, *FAP*, *CXCL1*, *CXCL2*, *CXCL3*, *CXCL5*, *CXCL6* and *CXCL8* were also significantly increased in M4/M5-high compared to M4/M5-low and -intermediate tissues, whereas *ABCA8* expression was reduced (Supplementary Table 8). Nevertheless, ABCA8 fibroblasts and PDGFRA fibroblasts both still expressed the above-mentioned chemokines in the M4/M5-intermediate and -high groups (Fig. 5b), raising the possibility that the inflammatory fibroblast cluster represents an activation state of ABCA8 fibroblasts and/or PDGFRA fibroblasts. Indeed, trajectory (pseudotime) analysis indicated that inflammatory fibroblasts may represent a transcriptomic state between ABCA8^+^ and PDGFRA^+^ fibroblasts (Extended Data Fig. 7f,g), thus potentially arising from either population.

In situ staining confirmed that tissues with dense neutrophil infiltrates (NE^+^ cells) exhibited the highest level of PDPN on fibroblasts, particularly in areas of profound epithelial cell loss (that is, ulceration) (Fig. 5c). This was associated with the expansion of THY1^+^ perivascular cells and blood endothelial vessels (PECAM1^+^THY1^+^PDPN^–^) (Fig. 5c). Furthermore, colocalization staining revealed that PDPN^+^ fibroblasts expressing FAP (magenta) are found in areas of NE^+ ^neutrophil influx (Fig. 5d).

### Neutrophil recruitment is driven through fibroblast IL-1R signaling

**Extended Data Fig. 8:**
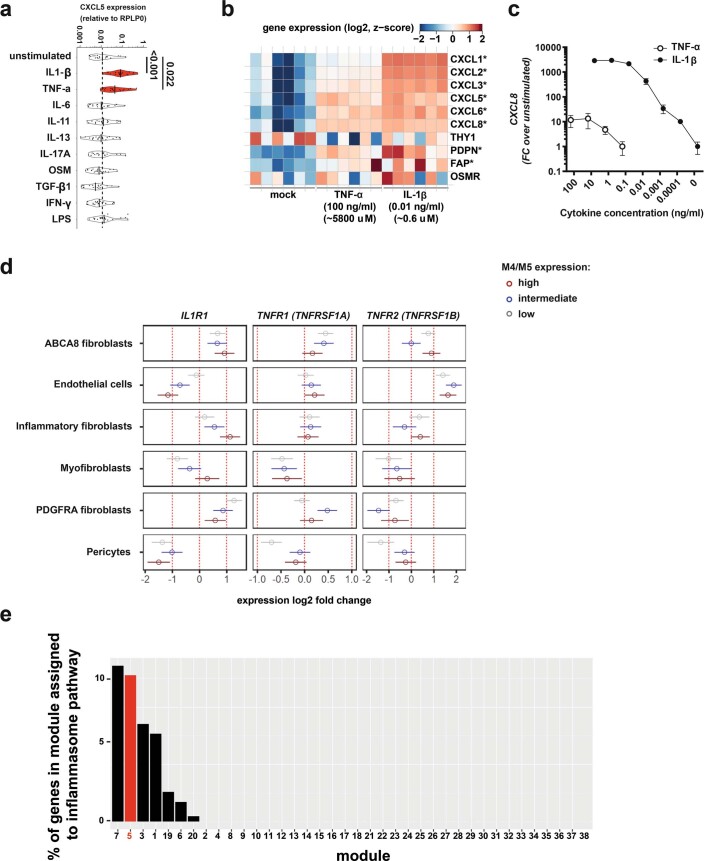
Cytokine response of intestinal fibroblasts. a) Primary fibroblast cell lines (n = 33) culture-expanded from resected IBD patient tissue and stimulated for 3 h with recombinant cytokines (adjusted P-values are shown where significantly different (p < 0.05) compared to unstimulated, Kruskall-Wallis test). b) RNAseq analysis (Salmon log2-transformed TPM values, z-score) of cultured intestinal fibroblast cell line Ccd18-co, stimulated with either TNF-α (100 ng/ml) or IL-1β (0.01 ng.ml) for 3 hours (n = 6 replicates per group, * P adjusted < 0.05 from DESeq2 differential gene expression analysis). c) Dose response of IL-1β and TNF-α stimulated Ccd18co fibroblasts for gene expression fold change (FC) of *CXCL8* over unstimulated, measured by qPCR; data are presented as mean values (n = 2) + /- SD. d) Pseudo-bulk expression fold changes (relative to M4/M5 low groups) of *ILR1* and *TNFR1* (see Supplementary Table 7) within the cellular clusters detected as in Fig. 3a, across patients with either low, intermediate or high M4/M5 whole tissue expression. e) Gene set enrichment analysis of all modules detected in the discovery cohort for genes assigned to inflammasome pathways (GO:0061702).

To identify upstream cytokine drivers of the neutrophil-attractant program in fibroblasts, we stimulated primary stromal cell lines expanded from surgically resected tissue from patients with IBD with a panel of disease-associated cytokines^4^. Of these, only the NF-kB activators IL-1β and TNF-α, but not IL-6 or OSM, induced *CXCL5* expression after 3 h of stimulation (Extended Data Fig. 8a). Furthermore, RNA-seq showed that IL-1β and TNF-α induced strong expression of genes encoding neutrophil-tropic CXCR1 and CXCR2 ligands in fibroblasts, namely *CXCL1*, *CXCL2, CXCL3*, *CXCL5*, *CXCL6* and *CXCL8* (Extended Data Fig. 8b). In addition, both cytokines induced the inflammatory fibroblast markers *PDPN* and *FAP* (Extended Data Fig. 8b). Although TNF-α and IL-1β both induced a chemokine response, the latter was 100-fold more potent (Extended Data Fig. 8b,c).

**Fig. 6:**
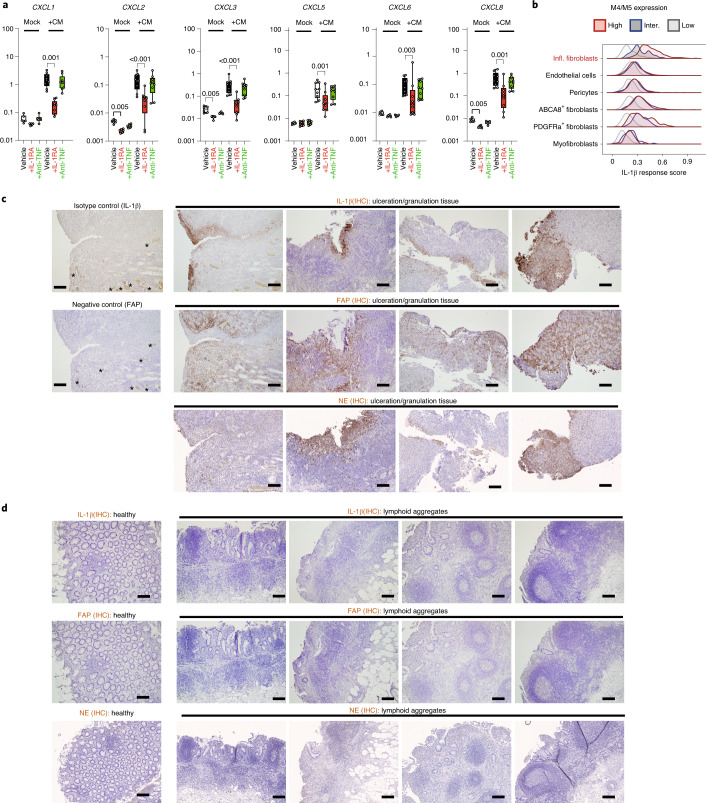
Activated inflammatory fibroblasts drive neutrophil recruitment through IL-1R signaling with high levels of IL-1β at sites of ulceration. **a**, Ccd18-Co fibroblasts were stimulated for 3 h with either mock control or conditioned media produced from *n* = 9 tissue digests from patients with IBD (CM), without pretreatment (vehicle, PBS) or preincubated with IL-1Ra (anakinra) or anti-TNF (adalimumab). Adjusted *P* values are shown where significant (*P* < 0.05); two-tailed Friedman test for paired samples. **b**, Projection of the IL-1 cytokine stimulation response of Ccd18-Co fibroblasts onto stromal cell clusters detected by scRNA-seq (Fig. 4a). Scores were computed as mean *z*-score of IL-1 upregulated genes. **c**, IHC staining of IL-1β, FAP or NE (DAB, counterstain hematoxylin) in *n* = 8 different inflamed (infl.) tissue sections of patients with IBD and with prominent ulceration and/or granulation tissue, and in *n* = 1 healthy tissue. Scale bars, 200 µm. * Indicates nonspecific staining of erythrocytes or platelets in vessels. **d**, Staining as in **c**, but of inflamed tissue sections from patients with IBD and with dominant lymphoid aggregates. Scale bars, 200 µm.

We used an ex vivo assay of conditioned medium (CM) generated from patients with IBD to confirm that IL-1 signaling could induce the inflammatory fibroblast phenotype (Fig. 6a). We blocked IL-1 signaling with the IL-1 receptor (IL-1R) antagonist anakinra (Kineret) or TNF signaling with the anti-TNF agent adalimumab (Humira) in CM. Strikingly, only IL-1R, but not TNF, blockade was able to reduce fibroblast activation in this assay (Fig. 6a). This demonstrates that soluble mediators derived from gut-resident cell populations of inflamed IBD tissue activate the neutrophil-attracting fibroblast program in an IL-1R- but not TNF-dependent manner.

Single-cell sequencing showed that inflammatory fibroblasts in M4/M5-high tissue from patients with IBD represented the cell population demonstrating the strongest IL-1 response signature (Fig. 6b; see Supplementary Table 9 for IL-1 gene expression response), suggesting that activation of this pathway may be associated with the poor therapy response observed in those patients. In support, inflammatory fibroblasts and ABCA8 fibroblasts demonstrated the highest fold increase of IL-1 receptor gene expression (*IL1R1*) in patients with M4/M5-high IBD (Extended Data Fig. 8d). By contrast, the expression of genes encoding TNF receptors (*TNFR1* and *TNFR2*) did not demonstrate this trend (Extended Data Fig. 8d). Consistent with the predominant role of IL-1 in these patients, module M5 was enriched in inflammasome genes (Extended Data Fig. 8e). Additionally, immunohistochemical staining revealed that IL-1β is localized to the ulcer bed and granulation tissue (Fig. 6c, top), but not to noninflamed tissue or that where lymphoid aggregates predominate (Fig. 6d, top). Areas of intense IL-1β labeling also demonstrated intense staining of FAP (Fig. 6c,d, middle), suggesting that IL-1R signaling in the ulcer bed drives the inflammatory fibroblast program characterized by FAP expression. Areas of IL-1β and FAP labeling also demonstrated infiltrates of NE^+^ neutrophils (Fig. 6c,d, bottom).

Overall, these results identify IL-1R signaling as a key driver of the inflammatory fibroblast/neutrophil recruitment phenotype that is observed in IBD tissues with a high M4/M5 pathotype.

## Discussion

Our results identify distinct pathotypes within the heterogeneous landscape of IBD that inform on the outcome of therapies. Several genes found in our M4/M5 modules have been associated with nonresponse to anti-TNF therapy or corticosteroids^5–7,9–12,15,22^, but previous studies failed to link these to the identifiable histopathological hallmarks, neutrophil contribution and cytokine drivers of this wider signature. These are important insights with implications for patient stratification in therapeutic targeting.

Our results highlight neutrophils as a major component of the M4/M5 signature associated with nonresponse to several different therapies in IBD. Previous analyses of tissue-level gene expression had linked neutrophils with therapy nonresponse in IBD^9,10^. We have extended those findings by mapping these signatures to intestinal neutrophils isolated from distinct tissue niches within lesions of inflamed intestine. It is also notable that a dominant neutrophil contribution to the biology of inflammation and anti-TNF therapy resistance in IBD is absent from previous scRNA-seq studies where these cells were not analyzed^6,7^. It is not known whether neutrophil accumulation is a cause or a consequence of the damage at sites of tissue ulceration. However, there is evidence that neutrophils can contribute to chronic inflammation through production of extracellular traps and the liberation of reactive-oxygen species^23^. We found that neutrophils are also the major source of *OSM* expression, a cytokine functionally associated with nonresponse to anti-TNF therapy in IBD^5^.

In situ localization of inflammatory fibroblasts by PDPN and FAP revealed their proximity to neutrophils in ulcers. We hypothesize that, rather than being a specialized fibroblast subset, inflammatory fibroblasts may represent an activation state of either ABCA8 fibroblasts residing in the lamina propria of the intestine, or subepithelial PDGFRA fibroblasts.

We also observed an expansion of ACKR1^+^ endothelial cells alongside activated fibroblasts in M4/M5-high tissues/deep ulcers. Module M2 also strongly correlated with endothelial cells in our in silico predictions, and was the third strongest association with therapy nonresponse. Since endothelial cells did not express high levels of genes coding for neutrophil-chemoattractant CXCLs, we can speculate that inflammatory fibroblasts produce these chemokines, which are then presented by ACKR1^+^ endothelial cells to circulating neutrophils to initiate extravasation^24^.

The very specific localization of IL-1β in the areas of epithelial cell damage in ulcers suggests that disruption of the epithelial barrier may be a primary event. Early responders to this damage may also include resident MNP, which can produce excessive IL-1β and IL-23 particularly in the context of IL-10 pathway deficiency^11^. Alarmin IL-1α may similarly contribute to the activation of fibroblasts and initiation of colitis^25,26^, and can be released by necrotic epithelial cells in IBD^27^. Further studies are required to establish whether the IL-1R-driven activation of inflammatory fibroblasts identified here is dominated by IL-1α or IL-1β signaling or both.

In addition to the M4/M5-high pathotype, we also identified patients with high M6 tissue expression associated with lymphoid aggregates. The high tissue expression of *CCL19*/*CCL21*/*CXCL13* suggests that this pathotype reflects the presence of fibroblastic reticular-like cells^20^. The expression of M6 was very low in pediatric-onset UC and CD; this may reflect the different nature of the samples analyzed in the two studies, the latter using endoscopic punch biopsies limited to the mucosa rather than full-thickness surgical specimens. This requires consideration when interpreting lymphoid signals from endoscopic biopsies.

Patients with UC or CD whose tissues show a high M4/M5 signature and ulceration express high amounts of *IL1B* but not *NR3C1*, *ITGA4 or TNF*, suggesting they may benefit from blocking of IL-1R rather than TNF to target the neutrophil-attractant program in fibroblasts. Indeed, TNF can promote mucosal healing^28^ and therefore may be deleterious in patients with deep ulceration. Genetic defects in the IL-1 pathway have been linked to anti-TNF nonresponse^29^, and the principle of ameliorating acute intestinal inflammation by blockade of IL-1 signaling has been demonstrated in preclinical models^30–32^. In case studies of Mendelian disease-like IBD with IL-10 deficiency, the blockade of IL-1 signaling can successfully treat intestinal inflammation^33,34^. Surprisingly, large-scale studies of IL-1 blockade in polygenic IBD patient cohorts are lacking although trials in acute severe ulcerative colitis are ongoing^35^.

Our surgical resection samples highlight the heterogeneity of inflammatory lesions in a difficult-to-treat patient group. These data are only a snapshot and do not inform on the evolution and dynamics of the distinct pathotypes identified here. However, the presence of M4/M5-signature-high patients before treatment in a number of prospective cohorts suggests that deep ulceration and high M4/M5 signature can occur independently of therapy failure. Our study does not address whether lymphoid aggregates and ulceration are independent processes or connected states. Notably, the presence of M4/M5 and M6 is not mutually exclusive, and a small number of tissues exhibited both ulceration and lymphoid aggregates. Further understanding of the natural history of these distinct pathotypes and their relationship to disease dynamics will require longitudinal analyses.

In summary, integration of data across biological levels identifies new tissular IBD pathotypes that are defined by different molecular, cellular and histopathologic features and are associated with patient responses to current therapeutics. Currently, subcategories of IBD are classified by high-level phenotypes. A deficit in understanding the cellular and molecular pathotypes in IBD has restricted prescription of therapeutics based on the underlying biologic processes they target, increasing the likelihood of failure. Future trials should consider patient inclusion criteria based on the observations presented here. Our data indicate that IL-1 blockade may benefit those individuals with deep ulceration and who do not respond to several different current therapeutics. This may improve treatment outcomes for patients with IBD by hastening the administration of appropriate interventions and providing an alternative therapeutic target in an area of unmet clinical need.

## Methods

### Statistics and reproducibility

Due to the nature of the study, no statistical method was used to predetermine sample size and experiments were not randomized; *n* = 15 samples of bulk sequencing from surgical resections were excluded based on failed quality control (low number of reads, outlier on PCA) (see GSE166928 for flags and https://github.com/microbialman/IBDTherapyResponsePaper). Given the sample nature, technical replication of experiments involving human patient sample material was not carried out because collection of substantially more tissue could not be ethically supported. Patient data were analyzed at the cohort level, using all patients/samples/datapoints, to derive statistics. For experiments with cell lines, replicates are presented and experiments shown only if the experiment was successfully repeated on at least one other occasion. Randomization was not relevant to the clinical findings because this was an observational study. Cell culture wells were randomly allocated to experimental groups. All human samples and cell line treatment groups were blinded to the researchers before data collection, by giving them a unique ID number. Pathologists were blinded for scoring of histopathologic slides. The type of statistical test and experimental group sizes are given in the figure legends.

### Patient cohorts and ethics

Patients eligible for inclusion in the discovery cohort were identified by screening surgical programs at Oxford University Hospitals. Samples were obtained from patients undergoing surgical resection of affected tissue for UC, CD or CRC (used as non-IBD controls). All tissue samples included in the study were classified by pathological examination as either macroscopically active inflamed or noninflamed. Additional samples were also obtained from patients with CD and UC, or from healthy individuals by biopsy. All patients and healthy participants gave informed consent and collection was approved by NHS National Research Ethics Service under the research ethics committee reference nos. IBD 09/H1204/30 and 11/YH/0020 (for IBD) and GI 16/YH/0247 (for CRC samples and gut biopsies from healthy individuals). Samples were immediately placed on ice (RPMI 1640 medium) and processed within 3 h. All data were fully anonymized before analyses. For replication of prospective findings in the discovery cohort, public datasets derived from endoscopic tissue samples of patients with IBD were used^10,15–17,36^ (GSE16879, GSE73661, GSE109142, GSE57945, GSE100833).

### Isolation of cells from tissue samples

After removal of external muscle and adipose layers, and removal of bulk epithelial cells by repeated washes in PBS containing antibiotics (penicillin/streptomycin, amphotericin B, gentamicin, ciprofloxacin) and 5 mM EDTA (Sigma-Aldrich), tissue from surgical resections was minced using surgical scissors. In the case of endoscopic biopsies, the epithelial wash was omitted. Minced tissue was subjected to multiple rounds of digestion in RPMI 1640 medium containing 5% fetal bovine serum (FBS), 5 mM HEPES, antibiotics as above and 1 mg ml^–1^ Collagenase A and DNase I (all Sigma-Aldrich). After 30 min, digestion supernatant containing cells was removed, filtered through a cell strainer, spun down and resuspended in 10 ml of PBS containing 5% bovine serum albumin (BSA) and 5 mM EDTA. Remaining tissue was then topped up with fresh digestion medium until no more cells were liberated.

### Primary culture expansion and conditioned medium production

Primary stromal cell lines were expanded by plating the single-cell suspension of tissue digests onto plastic cell culture vessels and expanding the adherent fraction in RPMI 1640 (with 20% fetal calf serum (FCS), antibiotics, 5 mM HEPES) (Sigma). Primary cell lines were used for assays between passage numbers 7 and 15. For the production of conditioned medium, sorted cell populations were plated at 1,000,000 cells ml^–1^ in cell culture dishes with RPMI 1640 containing 5% FCS (Life Technologies), antibiotics and 5 mM HEPES for 16 h. Next, supernatants were aspirated, spun down to remove cells and frozen at −80 °C until further use.

### FACS and analysis

Single-cell suspensions obtained from tissue digests were blocked in PBS 5% BSA containing human anti-Fc block (Miltenyi), except when CD16 was in the panel, stained for FACS analysis or sorting with Fixable Viability Dye eFluor 780 (eBioscience, 1:1,000), DAPI (Invitrogen, 1:50,000) and antibodies (all from Biolegend, except anti-Pdpn: clone NZ-1.3 from eBioscience and anti-FAP:sheep from Biotechne) in PBS with 5% BSA and 5 mM EDTA for 20 min on ice. After washing in the same buffer, cells were either analyzed (LSRII or Fortessa X20) or sorted (Aria III, 100-µm nozzle). All antibodies used for FACS analysis and sorting can be found in Supplementary Table 10.

### Ex vivo and in vitro assays of fibroblast stimulation

For the stimulation of stromal cells, either Ccd18-Co colonic fibroblasts (ATCC, no. CRL-1459) or primary stromal cell lines (isolated as above) were plated at 20,000 cells per well in a 48-well plate. Plated cells were starved for 72 h in culture medium without FCS, before stimulation with cytokines or conditioned medium (prediluted 1:3 in starving medium) for 3 h or 24 h at 37 °C. For blockade experiments, recombinant cytokines in starving or conditioned medium were preincubated with 2 mg ml^–1^ Anakinra (Kineret) or Adalimumab (Humira) for 1 h at room temperature, with shaking, before stimulation of cells. After 3 h, supernatants were removed and cells lysed directly in appropriate RNA lysis buffer.

### Isolation of RNA from tissue samples and cell populations

Endoscopic punch biopsies or dissected tissue pieces from surgical resections were stored in RNAlater (Qiagen) following collection, until further processing. Tissue was homogenized using the soft tissue homogenizing CK14 kit (Precellys, Stretton Scientific, no. 03961) in 300 µl of RLT lysis buffer (Qiagen) and 20 µM DTT (Sigma). RNA was isolated using the Qiagen Mini kit with a DNA digestion step (Qiagen). Bulk-sorted cell populations and cultured cells were directly lysed in RNA lysis buffer, followed by RNA isolation with the appropriate kits and on-column DNase treatment.

### Quantitative real-time PCR

Normalized amounts of isolated RNA were reverse transcribed using a high-capacity reverse transcription kit (Thermo Scientific, no. 4368814). Quantitative real-time PCR (qPCR) was performed using premade, exon-spanning Taqman probes (LifeTechnologies) and run on a ViiA 7 Real-Time PCR system. Gene expression values, relative to the housekeeping gene(s) as indicated, were calculated using the 2^Δct^ method.

### Sequencing of RNA from whole tissue and sorted cell populations

Sequencing libraries were prepared using either the QuantSeq 3’ mRNA-Seq FWD Library Prep Kit (Lexogen) for whole-tissue samples, or the Smart-seq2 protocol^37^ for bulk and cultured cell populations (with our own in-house indexing primers). Libraries were sequenced using an Illumina HiSeq4000 with 75-base-paired-end sequencing^38^. For qPCR analysis, 15–250 ng of RNA was reverse transcribed using the High-Capacity cDNA Reverse Transcription Kit (Applied Biosystems) and qPCR performed using Precision Fast qPCR mastermix with the EagleTaq Universal Master Mix at a lower level, 12.8 ml (Primer design, Precision FAST-LR), and Taqman probes (Life Technologies).

Bulk RNA sequencing data were analyzed using the bulk processing aspect of pipeline_scrnaseq.py (https://github.com/sansomlab/scseq). Data quality was assessed using pipeline_readqc.py (https://github.com/cgat-developers/cgat-flow). Sequenced reads were aligned to the human genome GRCh38 with Hisat2 (v.2.1.0)^39^ using a reference index built from the GRCm38 release of the mouse genome and known splice sites extracted from Ensembl v.91 annotations (using the hisat2_extract_splice_sites.py tool). A two-pass mapping strategy was used to discover new splice sites (with these additional parameters: –dta and–score-min L,0.0,−0.2). Mapped reads were counted using featureCounts (Subread v.1.6.3; Ensembl v.91 annotations; with default parameters)^40^. Salmon v.0.9.1 was used to calculate transcripts per million (TPM) values^41^ using a quasi-index (built with Ensembl v.91 annotations and *k* = 31) and gc bias correction (parameter ‘—gcBias’). For heatmap visualization of gene expression levels, *z*-scores of TPM values and Manhattan distances were calculated within the *heatmap2* package in R. Differential expression analyses were performed using DESeq2 (v.1.26.0)^42^.

Pathway enrichment analysis for groups of genes associated with cell types was carried out with the *enrichGO* function from the *clusterProfiler* package in R^43^. ‘Cellular component’ Gene Ontology annotation terms were used as pathways.

### Identification and quantification of gene coexpression modules in discovery data

To reduce dimensionality within the dataset, an unbiased approach was used to collapse genes with similar expression patterns in the discovery RNA-seq dataset. Normalized (TPM) counts were considered for all genes across all samples, including both inflamed and noninflamed tissues from patients with IBD and samples from the CRC controls. These were filtered to remove genes with zero counts in over half of the samples, and log transformed following the addition of a pseudocount. Transformed counts were then used to define modules of correlated genes using WGCNA in R^44^. In brief, this process calculates pairwise Pearson correlation estimates between all genes; these are then raised to the power of a soft threshold, in this case raising correlation coefficients to the power of 9, which magnifies the differences between large and small correlations. Finally, the network of these amplified correlations (where each gene is a node and each edge is a correlation) is used to generate a topological overlap matrix (TOM). This represents the similarity of expression patterns between a given pair of genes in the dataset, similar to the correlation matrix but taking into account their shared correlation with other genes. Finally, hierarchical clustering of the TOM is used to assign genes into modules based on their coexpression pattern. The *pickSoftThreshold* function was used to identify nine as an appropriate soft threshold. The *blockwiseModules* function was then used with this threshold to automatically carry out the aforementioned process and assign genes to modules. Parameters for the function were as follows: minimum module size of 30 genes, mergeCutHeight of 0.1, reassignThreshold of 0 and use of a signed network.

The resultant module definitions were quantified using the eigengene approach within WGCNA. An eigengene is a quantitative representation of the expression of a module as a whole, and is derived from the first component of a principle components analysis restricted to the expression data of only genes in the module. Eigengenes for the modules defined in the resection data were calculated using the *moduleEigengenes* function.

Correlations between clinical and metadata measures and module eigengenes were assessed using Pearson correlations, with *P* values estimated using the *corPvalueStudent* function and adjusted for multiple testing. Benjamini–Hochberg correction using the *p.adjust* function was used for all analyses with adjusted *P* values. This was carried out on inflamed IBD tissue samples and CRC tissue samples combined, and also on inflamed IBD tissue samples alone. Eigengenes were also compared between paired inflamed and noninflamed tissues sections using a *t*-test and adjusting for multiple testing across modules.

Cell type composition scores were estimated for each resection sample using the *xCellAnalysis* function from the xCell package^14^. Correlations between module eigengenes and the derived cell type scores were visualized for all cell types scored in >25% of samples, and used to infer the cell types represented by modules within the whole-tissue data (discovery cohort).

### Quantification of module associations with clinical variables in replication datasets

Publicly available RNA-seq^10,17^ (GSE57945, GSE109142) or microarray data^15,16,36^ (GSE16879, GSE12251, GSE100833) were downloaded from the NCBI gene expression omnibus. These were pre-existing enumerated gene counts in the case of the RNA-seq datasets and raw array data in the case of the microarray sets. The latter were processed and normalized to gene counts using the *rma* function from the affy package^45^, summing values for probes associated with the same gene symbol. Across all datasets, gene symbol annotations were used to map genes to the module assignments generated from the discovery resection tissue dataset, eliminating those not observed in the replication dataset under consideration. The percentage of genes missing from the original module definitions was recorded, but was generally low across all datasets. Mapped module assignments were then used to generate eigengenes from the replication expression datasets using the *moduleEigengenes* function. Correlations between clinical metadata and eigengenes in replication datasets was performed using Pearson correlations as for the discovery dataset. In the case of the pediatric cohort data (GSE57945), Mann–Whitney *U*-tests were used to compare modules between patients scored as ulcerated or not in metadata, and hierarchical clustering was used to group patients based on M4, M5 and M6 expression as for the discovery cohort.

Differences in pretreatment module eigengene values between responders and nonresponders in prospective studies were assessed using Mann–Whitney *U-*tests, adjusting *P* values for testing of multiple modules within each dataset. In the study of Haberman et al.^10^, we considered only patients on corticosteroid therapy, combining patients that received oral and intravenous administration. In the case of the 2018 study of Arjis et al.^16^, which tested multiple different therapies and treatment regimens, we used analysis of variance (ANOVA) to identify any differences between responders and nonresponders across all combinations, adjusting for regimen, and post hoc Mann–Whitney *U*-tests to identify individual treatment regimens where modules were significantly different by response.

Meta-analysis of the expression of modules M4 and M5 across responders and nonresponders in the various replication datasets was carried out using the meta package in R^46^. Anti-TNF response data were used from the Arijs 2009^15^ and 2018^16^ papers, and corticosteroid response data were from the study of Haberman et al. Only the 4-week treatment condition was included from the anti-intergrin data from ref. ^16^, as this was the only one that proved significantly different for either M4 or M5. A random effects meta-analysis was carried out comparing standardized mean differences between patient groups using the exact Hedges estimate.

In the prospective cohorts, the predictive value of the expression of single genes for response to treatment was assessed using a simple logistic regression where response was the outcome and gene expression the sole predictor. Modeling was carried out for all genes also observed in the discovery cohort, for each of the prospective studies, using the *glm* function in R. The predictive ability of each gene in each dataset was summarized as the area under the curve (AUC) of a receiver operating characteristic (ROC) curve. AUC values for each gene were generated by applying the *roc* function from the pROC package to predictions generated from the logistic regression models. The relative predictive power of genes within modules of interest was compared by summing the rank of genes (based on their AUC value) across datasets and comparing these cumulative ranks between modules.

### Pathological scoring of histology using the Nancy index

Formalin-fixed paraffin-embedded (FFPE) and hematoxylin-and-eosin-stained tissue sections of IBD patients were scored according to the Nancy index, based on criteria reported in ref. ^13^. The extent of histologic ulceration was scored on a semiquantitative scale (0–25–50–75–100% of section area). All scorings were carried out by blinded, consultant gastrointestinal histopathologists.

### Immunohistochemistry and quantitative histopathology

Tissue specimens were either fixed for 48 h in 4% neutral-buffered formalin (Sigma) and embedded in paraffin (FFPE) for chromogenic stainings, or fixed for 24 h in 2% paraformaldehyde in phosphate buffer containing l-lysine and sodium periodate and frozen in OCT (Sigma) after soaking in 30% sucrose for 48 h (OCT) for fluorescent staining. Freshly cut, dewaxed and rehydrated FFPE sections (5 µm) were subjected to heat-induced antigen retrieval by boiling in Target Retrieval Solution (Dako, pH 6.0, for all stainings except neutrophil elastase) for 15 min (microwave). This was followed by 15 min of blocking in Bloxall solution (Vector Labs), 60 min of blocking in 5% BSA/TBST with 5% serum of secondary antibody species (Sigma) and 15 min of blocking in avidin followed by biotin solution (Vector Labs). All steps were performed at ambient temperature. Tissue sections were incubated with primary antibodies in 5% BSA/TBST overnight (>16 h) at 4 °C. Following incubation, biotinylated or HRP-conjugated secondary antibodies were applied for 2 h (room temperature) in 5% BSA/TBST. For biotinylated secondary antibodies, AB complex (Vector Labs) was incubated for a further 1 h in TBST (room temperature). Chromogenic stains were developed by the application of DAB HRP substrate solution (Vector Labs) and counterstained for 5 min in hematoxylin solution (Sigma). Slides were then dehydrated and mounted in DPX (Sigma) mounting medium.

Whole-section imaging of chromogenic sections was performed on a NanoZoomer S210 digital slide scanner (Hamamatsu). Slide scans of all stains can be made available upon request. Scanned tissue sections, stained using DAB immunohistochemistry, were analyzed using Indica Labs HALO image analysis platform. A consultant gastrointestinal pathologist manually annotated each slide, dividing the mucosa into normal and inflamed areas. The tissue was scored using Indica Labs’ analysis modules CytoNuclear v.2.0.5, detecting DAB-positive and -negative cells in inflamed areas. Pathologic features (ulceration/granulation tissue, granulomas, crypt abscess/cryptitis, lymphoid aggregates and architectural distortion/mucin depletion) were manually annotated by a consultant pathologist with a special interest in gastrointestinal pathology. The area of each annotated feature was automatically calculated using HALO software. Nuclei (cells) in areas of interest and whole-tissue sections were detected and counted using Indica Labs’ CytoNuclear v.2.0.9 analysis module. Scores (percentage) were normalized to the number of nuclei found within a pathological feature over the total number of nuclei detected in the whole-tissue section. These normalized counts were used to investigate Pearson correlations between features and correlations with module eigengenes.

OCT sections (10 µM thickness) were incubated in blocking buffer (PBS1X, 5% goat serum, 2% FCS and human FcBlock, Miltenyi) with unconjugated primary antibodies (PDPN, PDGFRa, ABCA8), conjugated primary antibodies (THY1, NE, PECAM1, MCAM) or biotinylated (FAP) overnight at 4 °C. The following day, either AF488 donkey anti-rat, AF647 donkey anti-goat, AF555 donkey anti-rabbit or strepatividin-AF568 was applied for 1 h at room temperature in blocking buffer. Finally, nuclei were stained with Hoechst 28332 (Life Technologies) for 15 min at room temperature in blocking buffer and mounted in ProlongGold mounting medium (Life Technologies) before imaging with the spectral detector of a Zeiss confocal LSM 880 microscope. Images were processed and converted to TIFF format in Image J. All antibodies used for immunohistochemistry can be found in Supplementary Table 10.

### Preparation of cells for scRNA-seq

Four pairs of biopsies from the same patient were pooled, minced and frozen in 1 ml of CryoStor CS10 (StemCell Technologies) at −80 °C, then transferred to liquid nitrogen within 24 h. Single-cell suspensions from these endoscopic biopsies were then prepared by thawing, washing and subsequent mincing of the tissue using surgical scissors. Minced tissue was then subjected to rounds of digestion in RPM 1640 medium (Sigma) containing 5% FBS (Life Technologies), 5 mM HEPES (Sigma), antibiotics as above and Liberase TL with DNAse I (Sigma). After 30 min, digestion supernatant was removed, filtered through a cell strainer, spun down and resuspended in 10 ml of PBS containing 5% BSA and 5 mM EDTA. Remaining tissue was then topped up with fresh digestion medium until no further cells were liberated from the tissue. Cells were then stained and FACS-sorted, as described above for live EPCAM^–^CD45^–^ cells, before being taken for microfluidic partitioning.

### 10X library preparation, sequencing and data analysis

Single-cell RNA-seq data was generated from disaggregated intestinal tissue sorted for CD45^–^EPCAM^–^ stromal cells. Viable cells were subjected to a standard droplet single-cell complementary DNA library preparation protocol. The experimental details for generation of cDNA libraries are described in a separate manuscript^47^. We demultiplexed FASTQ files for each 10X library using the Cell Ranger (v.3.1.0) mkfastq function^48^. We then mapped reads to the GRCh38 human genome reference using Kallisto^49^ (v.0.46.0) and quantified genes by cell-barcode UMI matrices with Bustools (https://github.com/BUStools/bustools) (v.0.39.0). For quantification we used gene annotations provided by Gencode^50^ (release 33), keeping only protein-coding genes and collapsing Ensembl transcripts to unique HGNC-approved gene symbols.

We filtered for potentially empty droplets and damaged cells by excluding droplets with <500 unique genes and libraries with >20% of reads assigned to mitochondrial genes. We pooled the resulting high-quality cells from each 10X library into a single cell by gene UMI matrix. We normalized for read depth with the standard log(CP10K) normalization procedure for gene *g* and cell *i*, where log CP10K_*gi*_ = normalized log counts-per-10,000 for gene *g* and cell *i*, *h* = index for gene *h*, and *Σ*_*h*_ UMI_*hi*_ = total number ofcounts observed for cell *i*:

$$\log {\mathrm{CP}}10K_{gi} = {{{\mathrm{log}}}}\left( {1 + 10^4 \times \frac{{{\mathrm{UMI}}_{gi}}}{{\mathop {\sum }\nolimits_h {\mathrm{UMI}}_{hi}}}} \right)$$

We performed PCA analysis on the top 2,000 most variable genes, identified with the VST method implemented in the Seurat^51^ R package. For PCA, we *z*-scored each variable gene and computed the top 30 eigenvectors and singular values with the truncated SVD procedure, implemented in the RSpectra (https://github.com/yixuan/RSpectra) R package. We defined PCA cell embeddings by scaling eigenvectors by their respective singular values. To account for potential batch effects in PCA embeddings, we modeled and removed the effect of the 10X library as identified using the Harmony algorithm. For Harmony^19^, we set the cluster diversity penalty parameter *θ* to 0.5 and used default values for all other parameters. We evaluated the effect of library mixing before and after Harmony using the local inverse Simpson’s index (LISI), described in the Harmony manuscript^19^. We evaluated the significance of change in LISI with a *t*-test, with degrees of freedom equal to the number of libraries minus 1. To visualize cells in two dimensions, we input the Harmonized PCs into the uniform manifold approximation and projection (UMAP) (arXiv:1802:03426 [stat.ML]) algorithm.

### Identification of marker genes within scRNA-seq

We performed joint clustering analysis on all scRNA-seq libraries using the cells’ harmonized PCA embeddings. With the 30-nearest-neighbor graph, we computed the unweighted shared nearest neighbor (SNN) graph and truncated SNN similarity values <1/15 to zero. We then performed Louvain clustering based on the R/C++ implementation from Seurat at resolution 0.3, resulting in eight clusters. We identified upregulated marker genes in each cluster using pseudobulk differential expression with negative binomial regression, implemented in the DESeq2 R package. For pseudobulk analysis, we collapsed cells from the same donor and cluster into one pseudobulk sample, summing the UMI counts from each cell. We then performed differential expression analysis on these pseudobulk samples, with the design *y* ~ 1 + *cluster*. This design assigns each gene an intercept term (that is, mean expression), a multiplicative offset for each cluster. We addressed the degeneracy of the design matrix by assigning a Gaussian prior distribution to the cluster effects (DESeq2 parameter βPrior=TRUE). The full results for this differential expression analysis are reported in Supplementary Table 6.

### Differential expression analysis of single-cell data by inflammatory status

We performed differential expression to associate genes with inflammation status within each single-cell cluster. We used DESeq2 on the pseudobulk samples described above, this time analyzing each cluster separately with the design *y* ~ 1 + *InflamStatus*. We treated InflamStatus as a random effect (DESeq2 parameter βPrior=TRUE) and recovered a mean multiplicative offset for each of the three inflammatory status categories.

### Single-cell gene-set enrichment scoring

Single-cell gene-set enrichment scores were computed for WGCNA modules and cytokine stimulation signatures using the same strategy. For each gene in the gene set, we computed *z-*scores (mean centered and unit variance scaled) of log(CP10K) normalized expression across all cells. We then summed the *z*-scores of genes in the gene set to compute a single gene-set score for each cell. This procedure is summarized in the formula below, used to compute the score *S*_*G,i*_ for gene set *G* and cell *i* using normalized expression *y*_*gi*_, gene mean *μ*, and gene standard deviation *σ*_*g*_:

$${\mathrm{score}}_{G,i} = \mathop {\sum }\limits_{g \in G} (y_{gi} - \mu _g)/\sigma _g$$

### Single-cell trajectory analysis

We performed trajectory analysis using the principal curve method, implemented in the princurve R package (https://www.jstor.org/stable/2289936). We fit a principal curve to all fibroblasts by inputting harmonized UMAP coordinates into the principal_curve function. This mapped fibroblasts to a nonlinear, one-dimensional space and assigned each cell a unique position, from 0 to 100, along this trajectory. For direct visualization of the abundance of each cluster along the trajectory, we plotted the relative density of each cluster along it. In these density plots, ABCA8^+^ fibroblasts grouped towards the beginning (position 32) of the trajectory, PDPN^+^ fibroblasts in the middle (position 59) and PDGFRA^+^ fibroblasts towards the end (position 82). This distribution along the trajectory is also reflected by the canonical markers of these populations. To visualize this, we discretized pseudotime by binning into 100 uniform-density windows, chosen so that each window has the same number of cells. We then plotted the scaled gene expression values of ABCA8, PDPN and PDGFRA, summarized by mean expression (point) and 95% confidence interval (line).

### Reporting Summary

Further information on research design is available in the Nature Research Reporting Summary linked to this article.

## Online content

Any methods, additional references, Nature Research reporting summaries, source data, extended data, supplementary information, acknowledgements, peer review information; details of author contributions and competing interests; and statements of data and code availability are available at 10.1038/s41591-021-01520-5.

## Supplementary information

Supplementary Information

Reporting Summary

Supplementary Table 1 WGCNA discovery cohort analysis.

Supplementary Table 2 WGCNA module replications.

Supplementary Table 3 Therapy response dataset analyses—differential module expression.

Supplementary Table 4 Therapy response dataset analyses—AUCs.

Supplementary Table 5 Differential gene expression: stroma, neutrophils and MNPs.

Supplementary Table 6 Marker gene scRNA-seq clusters.

Supplementary Table 7 Differentially expressed genes between scRNA-seq clusters within M4/M5 groups.

Supplementary Table 8 Differentially expressed genes between M4/M5 groups within scRNA-seq clusters.

Supplementary Table 9 Differentially regulated genes in IL-1β-stimulated intestinal fibroblasts.

Supplementary Table 10 List of antibodies used in the study.

## Data Availability

Bulk RNA sequencing has been deposited at GEO (GSE166928) and single-cell data at ImmPort (SDY1765; accessible with the next release scheduled for 10 September 2021). Additional data have been made available through a GitHub repository at https://github.com/microbialman/IBDTherapyResponsePaper. Due to their extensive size, image scans and raw data for FACS/qPCR assays are available from the corresponding author (F.M.P.) upon request. Publicly available RNA-seq (GSE57945, GSE109142) and microarray data (GSE16879, GSE12251, GSE100833) were downloaded from the NCBI gene expression omnibus. Publicly available RNA-seq (GSE57945, GSE109142) and microarray data (GSE16879, GSE12251, GSE100833) were downloaded from the NCBI gene expression omnibus.

## Code availability

All code used for analysis and generation of figures can be found in the GitHub repository at https://github.com/microbialman/IBDTherapyResponsePaper.
