# Supplementary material for: IL-1-driven stromal–neutrophil interactions define a subset of patients with inflammatory bowel disease that does not respond to therapies
Source: Nat Med. 2021 Oct 21;27(11):1970–81. doi: 10.1038/s41591-021-01520-5 (PMC8604730; doi:10.1038/s41591-021-01520-5)
Supplement: Supplementary file 1 — Supplementary Information [file 41591_2021_1520_MOESM1_ESM.pdf]

nature portfolio

https://doi.org/10.1038/s41591-021-01520-5

Supplementary information

IL-1-driven stromal–neutrophil interactions define a subset of patients with inflammatory bowel disease that does not respond to therapies

In the format provided by the authors and unedited

Full list of Oxford IBD Cohort Investigators members: Carolina Arancibia[8], Adam Bailey[8], Ellie Barnes[8], Elizabeth Bird-Lieberman[8], Oliver Brain[8], Barbara Braden[8], Jane Collier[8], James East[8], Lucy Howarth[8,11], Paul Klenerman[8], Simon Leedham[8], Rebecca Palmer[8], Fiona M. Powrie[1], Astor Rodrigues[8,11], Alison Simmons[8], Peter Sullivan[8,11], Jack Satsangi[8], Philip Allan[8], Timothy Ambrose[8], Jan Bornschein[8], Jeremy Cobbold[8], Emma Culver[8], Michael Pavlides[8], Simon P. Travis[8], Holm H. Uhlig[8], Alissa Walsh[8]

[8]Translational Gastroenterology Unit, NIHR Oxford Biomedical Research Centre, Oxford University Hospitals NHS Foundation Trust, John Radcliffe Hospital, Oxford OX3 9DU, United Kingdom

[11]Department of Paediatrics, John Radcliffe Hospital, Oxford OX3 9DU, United Kingdom
