# Supplementary material for: IL-1-driven stromal–neutrophil interactions define a subset of patients with inflammatory bowel disease that does not respond to therapies
Source: Nat Med. 2021 Oct 21;27(11):1970–81. doi: 10.1038/s41591-021-01520-5 (PMC8604730; doi:10.1038/s41591-021-01520-5)
Supplement: Supplementary file 2 — Reporting Summary [file 41591_2021_1520_MOESM2_ESM.pdf]

nature research

Corresponding author(s): Fiona Powrie

Last updated by author(s): 20/08/2021

Reporting Summary

Nature Research wishes to improve the reproducibility of the work that we publish. This form provides structure for consistency and transparency in reporting. For further information on Nature Research policies, see our Editorial Policies and the Editorial Policy Checklist.

Statistics

For all statistical analyses, confirm that the following items are present in the figure legend, table legend, main text, or Methods section.

n/a	Confirmed	
☐	☒	The exact sample size (*n*) for each experimental group/condition, given as a discrete number and unit of measurement
☐	☒	A statement on whether measurements were taken from distinct samples or whether the same sample was measured repeatedly
☐	☒	The statistical test(s) used AND whether they are one- or two-sided<br>*Only common tests should be described solely by name; describe more complex techniques in the Methods section.*
☐	☒	A description of all covariates tested
☐	☒	A description of any assumptions or corrections, such as tests of normality and adjustment for multiple comparisons
☐	☒	A full description of the statistical parameters including central tendency (e.g. means) or other basic estimates (e.g. regression coefficient) AND variation (e.g. standard deviation) or associated estimates of uncertainty (e.g. confidence intervals)
☐	☒	For null hypothesis testing, the test statistic (e.g. *F*, *t*, *r*) with confidence intervals, effect sizes, degrees of freedom and *P* value noted<br>*Give P values as exact values whenever suitable.*
☒	☐	For Bayesian analysis, information on the choice of priors and Markov chain Monte Carlo settings
☒	☐	For hierarchical and complex designs, identification of the appropriate level for tests and full reporting of outcomes
☐	☒	Estimates of effect sizes (e.g. Cohen's *d*, Pearson's *r*), indicating how they were calculated

Our web collection on statistics for biologists contains articles on many of the points above.

Software and code

Policy information about availability of computer code

Data collection	Bulk RNA sequencing : Illumina HiSeq4000; Single Cell RNAsequencing : Illumina NextSeq 500; Image acquisition : NanoZoomer S210 Hamatsu (NDP.view2 U12388-21) Zeiss Confocal LSM 880 (Zen System 3.4); FACS acquisition and sort : LSRII, Aria III (FACSDiva v8.0); quantitative PCR : ViiA 7 Real-Time PCR System (QuantStudio 6)
Data analysis	Bulk RNA sequencing data were analysed in Python using the bulk processing aspect of pipeline_scrnaseq.py (https://github.com/sansomlab/scseq). Data quality was assessed using pipeline_readqc.py (https://github.com/cgat-developers/cgat-flow). Sequence read were aligned using the hisat2_extract_splice_sites.py tool. Mapped Reads were counted using featureCounts (Subread version 1.6.3). Salmon v0.9.1 was used to calculate TPM values. R (v3.6.1) packages_version used for analysis were as follows:<br>rstatix_0.7.0; clusterProfiler_3.14.3; gmodels_2.18.1; simpleaffy_2.62.0; gcrma_2.58.0; genefilter_1.68.0; affy_1.64.0; knitr_1.33; biomaRt_2.42.1; annotate_1.64.0;XML_3.99-0.3;BiocManager_1.30.15; stringi_1.6.2;egg_0.4.5; gridExtra_2.3; gtools_3.8.2; ggrepel_0.9.1; GO.db_3.10.0; org.Hs.eg.db_3.10.0; AnnotationDbi_1.48.0; IRanges_2.20.2;S4Vectors_0.24.4;vroom_1.4.0; RColorBrewer_1.1-2;gplots_3.1.1;edgeR_3.28.1;limma_3.42.2;pROC_1.17.0.1;data.table_1.14.0;OptimalCutpoints_1.1-4;caret_6.0-88 ;lattice_0.20-38;ROCR_1.0-11;ROCit_2.1.1;ggpubr_0.4.0;WGCNA_1.70-3;fastcluster_1.1.25;dynamicTreeCut_1.63-Z;forcats_0.5.1;stringr_1.4.0;dplyr_1.0.6;purrr_0.3.4;readr_1.4.0;tidyr_1.1.3;tibble_3.1.2;ggplot2_3.3.3;tidyverse_1.3.1;GEOquery_2.54.1;Biobase_2.46.0;BiocGenerics_0.32.0; gplots_3.1.1,DESeq2_1.26.0;xCell_1.1.<br>Microarray from publicly available sets were processed and normalised using the affy package (v1.64.0). Meta-analysis of the expression of modules in the replication datasets was carried out using the meta package (v4.19.0) and AUC values for each gene were generated by applying the roc function from the pROC (v1.17.0.1) package in R.<br>Quantitative histopathology were carried out in Indica Labs HALO® image analysis platform using Indica Labs analysis modules CytoNuclear v2.0.5 and v2.0.9. Fluorescent image were analyzed and merged using imageJ software (v1.53c).<br>Single-cell sequencing data was processed using Cell Ranger (v3.1.0), Kallisto (v0.46.0) and Bustools (https://github.com/BUStools/bustools) (v0.39.0). PCA analysis was performed with the VST method implemented in the Seurat R package and R Spectra (https://github.com/yixuan/RSpectra). Batch effects in the PCA embeddings, was modelled and removed using the Harmony algorithm. Harmonized UMAP was generated with the UMAP (arXiv:1802:03426 [stat.ML]) algorithm. Data analysis was then performed using Seurat and Deseq2 and princurve R (https://

www.jstor.org/stable/2289936) packages in R software.
FACS analysis was performed using FlowJo v(10.7.1).
Figure visualisation and statistics analysis were performed using GraphPad v8.4.2, Adobe illustrator 2019 (v23.1) and R (v3.61 and 4.0.1).
All details and code relating to these analyses is available in the following Github repository: https://github.com/microbialman/IBDTherapyResponsePaper

For manuscripts utilizing custom algorithms or software that are central to the research but not yet described in published literature, software must be made available to editors and reviewers. We strongly encourage code deposition in a community repository (e.g. GitHub). See the Nature Research guidelines for submitting code & software for further information.

Data

Policy information about availability of data

All manuscripts must include a data availability statement. This statement should provide the following information, where applicable:

- Accession codes, unique identifiers, or web links for publicly available datasets
- A list of figures that have associated raw data
- A description of any restrictions on data availability

Bulk RNA sequencing has been deposited on GEO (GSE166928) and single-cell data on ImmPort (SDY1765; accessible with the next release scheduled for September 10 2021). Additional data has been made available through a GitHub repository at https://github.com/microbialman/IBDTherapyResponsePaper. Due to the extensive size, image scans, and raw data for FACS/qPCR assays are available from the corresponding author (FMP) upon request.
Publicly available RNAseq (GSE57945, GSE109142) or microarray data (GSE16879, GSE12251, GSE100833) were downloaded from the NCBI gene expression omnibus.

Field-specific reporting

Please select the one below that is the best fit for your research. If you are not sure, read the appropriate sections before making your selection.

☒ Life sciences ☐ Behavioural & social sciences ☐ Ecological, evolutionary & environmental sciences

For a reference copy of the document with all sections, see nature.com/documents/nr-reporting-summary-flat.pdf

Life sciences study design

All studies must disclose on these points even when the disclosure is negative.

Sample size	The number of available samples was dictated by the number of interventions (surgeries, endoscopies) carried out, of which we collected every possible sample. Conclusions in the manuscript relating to this data are supported by appropriate statistical tests, and where possible all datapoints are shown.
Data exclusions	n=15 samples of bulk sequencing from surgical resections were excluded based on failed quality control (low number of reads, outlier on PCA) (see GSE166928 for flags and https://github.com/microbialman/IBDTherapyResponsePaper)
Replication	Given the sample nature, technical replication of experiments involving human patient sample material was not carried out as collecting substantially more tissue could not be ethically supported. Patient data was analysed at a cohort level, using all patients/samples/datapoints, to derive statistically meaningful conclusions. For experiments with cell lines, replicates are presented and experiments only shown if the experiment could was successfully repeated at least one other occasion.
Randomization	Randomization was not relevant to the clinical findings as this was an observational cohort. Cell culture wells were randomly allocated to experimental groups.
Blinding	All human samples and cells lines used in this paper were blinded before data collection by giving them a unique ID number. Pathologists were blinded for scoring of histopathologic slides.

Reporting for specific materials, systems and methods

We require information from authors about some types of materials, experimental systems and methods used in many studies. Here, indicate whether each material, system or method listed is relevant to your study. If you are not sure if a list item applies to your research, read the appropriate section before selecting a response.

Materials & experimental systems

n/a	Involved in the study
☐	☒ Antibodies
☐	☒ Eukaryotic cell lines
☒	☐ Palaeontology and archaeology
☒	☐ Animals and other organisms
☐	☒ Human research participants
☒	☐ Clinical data
☒	☐ Dual use research of concern

Methods

n/a	Involved in the study
☒	☐ ChIP-seq
☐	☒ Flow cytometry
☒	☐ MRI-based neuroimaging

Antibodies

Antibodies used

Antibodies for FACS and IF analysis or sorting cells were from Biolegend (all used @1:200 dilution except for CD45, CD56, CD3, PDGFRA, CD19 @1:100, and CD4, CD8, Sig8 @1:50): BV785 antiCD45 (clone HI30 ref 304024, lot B284678), AF700 antiCD45 (clone HI30, ref 304048, lot B219921), APC antiHLADR (clone L243, ref 307610, lot B214996), BV711 antiHLADR (clone L243, ref 307644, lot B271297), PE antiCD14 (clone HCD14, lot B269397), BV421 antiCD14 (clone HCD14, ref 325628, lot B286544), PeCy5 antiCD3 (clone UCHT1, ref 300410, lot B258253), AF700 antiCD3 (clone SK7, ref 344828, lot B245810), Pedazzle antiCD3 (clone SK7, ref 344844, lot B220096), PEdazzle antiCD66b (clone G10F5, ref 305122, lot B279539), PerCpCy5.5 antiCD66b (clone G10F5, ref 305108, lot B260775), BV711 antiCD56 (clone 5.1h11, ref 362542, lot B327219), AF488 antiCD19 (clone HIB19, ref 302219, lot B238185), AF700 antiCD19 (clone HIB19, ref 30226, lot B254252), Pecy7 antiCD11c (clone S-HCL-3, ref 371508, lot B258655), BV510 antiCD16 (clone 3G8, ref 302048, lot B265264), PE antiSiglec8 (clone 7C9, ref 347104, lot B261537), Pedazzle antiCD15 (clone W6D3, ref 323038, lot B228060), PeCy7 antiCKIT (clone 104D2, ref 313212, lot B264778), BV510 antiCD4 (clone OKT4, ref 317444, lot B252921), AF700 antiCD8A (clone SK1, ref 344724, lot B282429), BV605 antiPECAM1 (clone WM59, ref 303122, lot B229211), AF647 antiPECAM1 (clone WM59, ref 303112, lot B161860), FITC antiEPCAM (clone 9C4, ref 324204, lot B261790), Pedazzle antiEPCAM (clone 9C4, ref 324232, lot B270029), Pedazzle antiTHY1 (clone 5E10, ref 328134, lot B266076), BF650 antiTHY1 (clone 5E10, ref 328144, lot B263262), PE antiPDGFRa (clone 16A1, ref 323506, lot B275570). eBioscience : PerCp-eFluor 710 antiPDPN (clone Nz1.3, ref 46-9381-42, lot 4332205) , APC antiPDPN (clone Nz1.3, ref 17-9381-42, lot 4341948). R&Dsystems : APC antiFAP (Clone 427819, ref FAB3715A-100, lot AEHI0119011).
Further antibodies used for IHC/IF studies were: AF488 antiNeutrophil Elastase @1ug/ml (clone NP57, company :Santacruz, ref sc-53388-AF488, lot G1019), antiPDPN @1:100 (clone D2-40, company :Dako, ref ISO72, lot 10095921), antiPDPN @5ug/ml (clone Nz1.3, company ;eBioscience, ref 17-9381-42, lot 2065602), antiTHY1 @5ug/ml (clone 5E10, biolegend, ref 328102, lot B216386), antiNeutrophil Elastase @1:100 (clone NP57, company Santacruz, ref sc-53388, lot : G0810), AF488 antiMCAM @5ug/ml (clone P1H12, company Biolegend, ref 361020, lot B270164), Biotinylated antiFAP @1:100 (polyclonal, company : R&D systems, ref AF3715), antiCD68 @1:500 (polyclonal, company : Sigma-Aldrich, ref HPA048982, lot 007002291), antiCalprotectin @1:100 (clone MAC387, company :Bio-Rad, ref MCA874G, lot : 1801), antiIL1b @1:100 (clone 3A6, company : Cellsignalling, ref : 12242S, lot :1), antiABCA8 1ug/ml (polyclonal, company : ThermoFisher, ref PA5-60866, lot A105833), antiPDGFRa @5ug/ml (polyclonal, company : R&Dsystems, ref AF-307-NA, lot VG0718081). Secondary antibodies included : Biotinylated anti-rabbit IgG @1:500 (company : Vectorlab, ref BA-1000, lot ZE1218), Biotinylated anti-mouse @1:500 (company : Vector lab, ref BA-2000, lot ZG0430), AF488 antiRat IgG @1:500 (company : Invitrogen , ref A-31572, lot 2017396), AF647 antiGoat @1:500 (company : invitrogen, ref A-21447, lot 1739289).
Isotype control included : AF647 mouseIgG1 (clone MOPC-21, company Biolegend , ref 400135, lot B241547), AF488 mouse IgG1 (clone MOPC-21, company :Biolegend, ref : 400129, lot B27964), AF488 mouseIgG1 (company: santacruz, ref sc-3890, lot K2619), Rat IgG2a (company :ebioscience, ref 16-4321-82, lot 2269842), mouse IgG1 (Company : SigmaAaldrich, ref 02-6100, lot TB266463)

Validation

FACS :
All antibobies from Biolegend have been validated by the manufacturer for use in flow cytometry. Additional information can be obtained on the company websites.
anti-PDPN , clone Nz1.3 Percp eFluor710 /APC have been validated by the manufacturer for flow cytometry. antiFAP APC antibody has been validated by the manufacturer for flow cytometry.
IHC :
Mouse anti S100A9 (Clone MAC387- Biorad), Rabbit polyclonal antiCD68 (Reference HPA048982 Sigma-aldrich), Mouse anti human NE (NP47- SantaCruz), Goat anti PDGFRA (Reference : AF-307-NA Biotechne), IL1beta (Clone 3A6 CellSignalling 12242S), mouse anti-PDPN antibody (Clone D2-40; Dako) were validated by manufacturer for use in IHC on paraffin.
Biotinylated sheep anti-FAP (Reference : AF3715, Biotechne) was validated in house for IHC on paraffin sections, normal adjacent CRC tissue was used as a negative control for antibody specificity.
IF:
Rat anti human PDPN (NZ 1.13- Ebioscience), biotinylated sheep anti-FAP (Reference : AF3715, Biotechne), Goat anti PDGFRA (Reference : AF-307-NA Biotechne), Rabbit anti ABCA8 (PA5-60866, ThermoFisher). -Conjugated, Mouse anti human THY1 (5E10, Biolegend), AF488 anti NE (NP57, Santa cruz) , AF647 anti PECAM1 (WM59, Biolegend), AF488 mouse antiMCAM (P1H12, Biolegend) were all validated in house for use in IF after PLP fixation. Unspecific binding was assessed with Isotype control.

Eukaryotic cell lines

Policy information about cell lines

Cell line source(s)

Ccd18-Co (ATCC, ref : CRL-1459) colonic fibroblast cell lines

Authentication

STR profiling by the vendor

Mycoplasma contamination	Cell lines tested negative for mycoplasma contamination on all occasions.
Commonly misidentified lines (See ICLAC register)	No commonly misidentified cell lines were used

Human research participants

Policy information about studies involving human research participants

Population characteristics	We analyzed 39 non IBD surgical resection from large and small intestine (54% Male, age median 68, 39% of large intestine), 31 IBD surgical resection from large and small intestine (25.8% UC, 70.9% CD, 3,22% IBDu, 52% Male, age median 37, 27% large intestine). Patients medications before surgery was analyzed as covariates and included 19% of aminosalicylates, 39% of corticosteroids, 68% of immunomodulators, 42% of antiTNFa antibodies, 7% of anti-integrin antibodies.<br>We also analysed intestinal biopsies sections from UC patients under anti-TNFa therapy. 35 patients were responding to treatment (65,7% Male, age median 32), 23 patients were not responding to antiTNFa (53.3% Male, age median 34).<br>Additionally, untestinal biopsies from IBD patients were used for FACS and sorted bulk RNA sequencing analysis including 9 UC and 3 CD patients (50% Male, age median 34).<br>4 Healthy biopsies and 7 UC biopsies were used for single cell RNA sequencing (100% Female, age median 47).
Recruitment	Patients were recruited through the Oxford TGU Biobank, UK. Intestinal biopsies were taken from patients followed up at the John Radcliffe hospital for their IBD management; healthy intestinal biopsies were taken from patients undergoing endoscopy for suspected intestinal symptoms, but where IBD/intestinal inflammation was not diagnosed. Surgical resection were obtained from surgeries scheduled as part of the disease management for patients with IBD or CRC . Patients analysed for single-cell RNAseq were exclusively female; no gender bias in IBD is reported, hence it can be assumed that gender is not a confounding factor in any of our analyses.
Ethics oversight	All patients and healthy participants gave informed consent and collection was approved by NHS National Research Ethics Service under the research ethics committee references IBD 09/H1204/30 and 11/YH/0020 for IBD or GI 16/YH/0247 for CRC samples and gut biopsies from healthy individuals.

Note that full information on the approval of the study protocol must also be provided in the manuscript.

Flow Cytometry

Plots

Confirm that:

☒ The axis labels state the marker and fluorochrome used (e.g. CD4-FITC).

☒ The axis scales are clearly visible. Include numbers along axes only for bottom left plot of group (a 'group' is an analysis of identical markers).

☒ All plots are contour plots with outliers or pseudocolor plots.

☒ A numerical value for number of cells or percentage (with statistics) is provided.

Methodology

Sample preparation	Minced tissue from intestinal biopsies or surgical resection was subjected to multiple rounds of digestion in RPMI1640 medium containing 5% fetal bovine serum (FBS), 5mM HEPES, antibiotics as above, and 1mg/ml Collagenase A and DNase I (all from Sigma Aldrich). After 30 minutes, digestion supernatant containing cells was taken off, filtered through a cell strainer (50uM), spun down and resuspended in 10ml of PBS containing 5% BSA and 5mM EDTA. Remaining tissue was then topped up with fresh digestion medium until no more cells were liberated from the tissue. Single-cell suspensions obtained from tissue digests were stained for FACS analysis or sorting with antibodies in PBS with 5% BSA and 5mM EDTA for 20 minutes on ice. After washing in the same buffer, cells for FACS analysis were fixed for 20min in PFA4% (BD fixative buffer) Sorted cells were not fixed but directly sorted in PBS with 5% BSA and 5mM EDTA buffer.
Instrument	FACS analysis was done on a LSRII (BD) , cells were sorted on an AriaIII sorter (BD)
Software	FACS data were analyzed in FlowJo software v(10.7.1)
Cell population abundance	Between 15000 and 5000 Immune and stromal cell were sorted for Extended Figure 5b. Between 10 000 and 100 000 cells were sorted for Bulk RNA sequencing (Figure 3c). The number of cells depended on the inflammation status of the tissue and the size of the surgical pieces removed. Cell purity was confirmed by FACS analyzing fraction of the sorted cells, all cells sorted only expressed the selecting markers. Gene expression of the different marker on which cells were sorted was also confirmed by RTqPCR on sorted pure populations.
Gating strategy	Cells were distinguished from debris based on FSC-SSC , single cells were isolated from doublets by FSC-H, FSC-W, live cells were distinguished from dead cells using negative selection for DAPI staining (Thermoscientific 62248) and Viability Dye EF780 (ebioscience , 65-0865-14).<br>Epithelial cells, Endothelial cells and Stromal cells were gated on CD45 negative event. EPCAM were then selected on EPCAM positivity, Endothelial cells on PECAM1 positivity. Stromal cells were identified as EPCAM, PECAM1 negative but THY1 and PDPN positive.<br>Leukocytes were gated on CD45 positivity.

CD8 T cells were then gated on CD3e and CD8 positivity, CD4 T cells were gated on CD3e and CD4 positivity. NK cells were gated on CD56 positivity. B cells were gated on CD3 and CD56 negativity and CD19 positivity.
Granulocytes were gated on CD3, CD19, HLADR negativity and CD66b positivity. Neutrophils were gated on CD15 and CD16 positivity. Mast cells were gated on CD15 and CD16 negativity and cKIT positivity. Eosinophils were gated CD15 and CD16 intermediate positivity and SIGLEC8 positivity.
Myeloid cells were gated on CD3, CD19 and CD56, CD66b negativity and HLADR positivity. MNP were then distinguished based on CD14 and CD11c expression.
All cells were back gated on FSC and SSC to confirm size and granularity.

☒ Tick this box to confirm that a figure exemplifying the gating strategy is provided in the Supplementary Information.
