# Supplementary material for: IL-1-driven stromal–neutrophil interactions define a subset of patients with inflammatory bowel disease that does not respond to therapies
Source: Nat Med. 2021 Oct 21;27(11):1970–81. doi: 10.1038/s41591-021-01520-5 (PMC8604730; doi:10.1038/s41591-021-01520-5)
Supplement: Supplementary file 12 — List of antibodies used in the study. [file 41591_2021_1520_MOESM12_ESM.pdf]

Methods	Antibody	Clone	Company	Reference	Lot number
Directly conjugated					
FACS	BV785/AF700 anti-CD45	HI30	Biolegend	304024 / 304048	B284678/B219921
FACS	APC/BV711 anti-HLADR	L243	Biolegend	307610 / 307644	B214996/B271297
FACS	PE/BV421 anti-CD14	HCD14	Biolegend	325606 / 325628	B269397/B286544
FACS	AF700/ PEdazzle anti-CD3	SK7	Biolegend	344828 / 344844	B245810/B220096
FACS	PeCy5 anti-CD3	UCHT1	Biolgend	300410	B258253
FACS	Pedazzle/ PerCpCy5.5 anti-CD66b	G10F5	Biolegend	305122 / 305108	B279539/B260775
FACS	BV711 anti-CD56	5.1h11	Biolegend	362542	B327219
FACS	AF488/ AF700 CD19	HIB19	Biolegend	302219 / 302226	B238185/B254252
FACS	PeCy7 anti-CD11c	S-HCL-3	Biolegend	371508	B258655
FACS	BV510 anti-CD16	3G8	Biolegend	302048	B265264
FACS	Pe anti-Siglec8	7C9	Biolegend	347104	B261537
FACS	Pedazzle anti-CD15	W6D3	Biolegend	323038	B228060
FACS	PeCy7 anti-cKIT	104D2	Biolegend	313212	B264778
FACS	BV510 antiCD4	OKT4	Biolegend	317444	B252921
FACS	AF700 antiCD8a	SK1	Biolegend	344724	B282429
FACS – IHC (OCT)	BV605 / AF647 anti-CD31	WM59	Biolegend	303122/ 303112	B229211/B161860
FACS	FITC/ Pedazzle anti-EPCAM	9C4	Biolegend	324204 / 324232	B261790/B270029
FACS	Pedazzle/ BV650 antiThy1	5E10	Biolegend	328134 / 328144	B266076/B263262
FACS	PE antiPDGFRa	16A1	Biolegend	323506	B275570
IHC (OCT)	AF488 antiNeutrophil Elastase	NP57	SantaCruz	sc-53388-AF488	G1019
FACS	Percp eFluor710 /APC antiPDPN	Nz1.3	eBioscience	46-9381-42 / 17-9381-42	4332205/4341948
FACS	APC antiFAP	427819	R&D systems	FAB3715A-100	AEHI0119011
IHC (OCT)	AF488 antiMCAM	P1H12	Biolegend	361020	B270164
Unconjugated					
IHC (FFPE)	antiPDPN	Clone D2-40	Dako	IS072	10095921
FACS-IHC (OCT)	antiPDPN	NZ1.3	eBioscience	17-9381-42	2065602
IHC (OCT)	Thy1	5E10	Biolegend	328102	B216386
IHC (FFPE)	Neutrophil Elastase	NP57	Santacruz	sc-53388	G0810
IHC (FFPE and OCT)	Biotinylated- antiFAP	Polyclonal sheep	Biotechne	AF3715	
IHC (FFPE)	CD68	Polyclonal rabbit	Sigma-aldrich	HPA048982	007002291
IHC (FFPE)	S100A8/9 (Calprotectin)	MAC387	Bio-Rad	MCA874G	1801
IHC (FFPE)	IL1beta	3A6	CellSignalling	12242S	1
IHC (OCT)	ABCA8	Polyclonal Rabbit	ThermoFisher	PA5-60866	A105833
IHC (OCT)	PDGFRa	Polyclonal Goat	Biotechne	AF-307-NA	VG0718081
Isotype Control					
IHC (OCT)	AF647mouse IgG1	MOPC-21	Biolegend	400135	B241547
IHC (OCT)	AF488 mouse IgG1	MOPC-21	Biolegend	400129	B27964

IHC (OCT)	AF488 mouse IgG1	-	Santacruz	Sc-3890	K2619
IHC (OCT)	Rat IgG2a	-	eBioscience	16-4321-82	2269842
IHC (OCT-FFPE)	Mouse IgG1	-	Sigma-Aldrich	02-6100	TB266463
Secondary antibody					
IHC (FFPE)	Biotinylated Anti-Rabbit IgG Antibody	Goat	Vector lab	BA-1000	ZE1218
IHC (FFPE)	Biotinylated Anti-Mouse antibody	Horse	Vector Lab	BA-2000	ZG0430
IHC (OCT)	Anti Rat IgG -AF488	Donkey	Invitrogen	A-21208	2180272
IHC (OCT)	Anti Rabbit IgG-AF555	Donkey	Invitrogen	A-31572	2017396
IHC (OCT)	Anti-Goat IgG AF647	Donkey	Invitrogen	A-21447	1739289
